# Size-advantage of monovalent nanobodies against the macrophage mannose receptor for deep tumor penetration and tumor-associated macrophage targeting

**DOI:** 10.7150/thno.77560

**Published:** 2023-01-01

**Authors:** Marco Erreni, Francesca D'Autilia, Roberta Avigni, Evangelia Bolli, Sana M. Arnouk, Kiavash Movahedi, Pieterjan Debie, Achille Anselmo, Raffaella Parente, Cécile Vincke, Fijs W.B. van Leeuwen, Paola Allavena, Cecilia Garlanda, Alberto Mantovani, Andrea Doni, Sophie Hernot, Jo A. Van Ginderachter

**Affiliations:** 1 Unit of Advanced Optical Microscopy, IRCCS Humanitas Research Hospital -, via Manzoni 56, 20089 Rozzano, Milan, Italy.; 2 Department of Biomedical Sciences, Humanitas University, Via Rita Levi Montalcini 4, 20090 Pieve Emanuele, Milan, Italy.; 3 IRCCS Humanitas Research Hospital -, via Manzoni 56, 20089 Rozzano, Milan, Italy.; 4 Cellular and Molecular Immunology Lab, Vrije Universiteit Brussel, Brussels, Belgium (Pleinlaan 2, 1050 Brussels).; 5 Myeloid Cell Immunology Lab, VIB Center for Inflammation Research, Brussels, Belgium.; 6 Laboratory for In vivo Cellular and Molecular Imaging (ICMI-BEFY/MIMA), Vrije Universiteit Brussel, Brussels, Belgium (Laarbeeklaan 103, 1090 Brussels).; 7 Leiden University Medical Center, Interventional Molecular Imaging Laboratory, Albinusdreef 2 2333 ZA Leiden.; 8 The William Harvey Research Institute, Queen Mary University of London, London EC1M6BQ, UK.

**Keywords:** tumor-associated macrophage targeting, macrophage mannose receptor, single-domain antibody, intravital microscopy, pharmacokinetics

## Abstract

**Rationale:** Nanobodies (Nbs) have emerged as an elegant alternative to the use of conventional monoclonal antibodies in cancer therapy, but a detailed microscopic insight into the *in vivo* pharmacokinetics of different Nb formats in tumor-bearers is lacking. This is especially relevant for the recognition and targeting of pro-tumoral tumor-associated macrophages (TAMs), which may be located in less penetrable tumor regions.

**Methods:** We employed anti-Macrophage Mannose Receptor (MMR) Nbs, in a monovalent (m) or bivalent (biv) format, to assess *in vivo* TAM targeting. Intravital and confocal microscopy were used to analyse the blood clearance rate and targeting kinetics of anti-MMR Nbs in tumor tissue, healthy muscle tissue and liver. Fluorescence Molecular Tomography was applied to confirm anti-MMR Nb accumulation in the primary tumor and in metastatic lesions.

**Results:** Intravital microscopy demonstrated significant differences in the blood clearance rate and macrophage targeting kinetics of (m) and (biv)anti-MMR Nbs, both in tumoral and extra-tumoral tissue. Importantly, (m)anti-MMR Nbs are superior in reaching tissue macrophages, an advantage that is especially prominent in tumor tissue. The administration of a molar excess of unlabelled (biv)anti-MMR Nbs increased the (m)anti-MMR Nb bioavailability and impacted on its macrophage targeting kinetics, preventing their accumulation in extra-tumoral tissue (especially in the liver) but only partially influencing their interaction with TAMs. Finally, anti-MMR Nb administration not only allowed the visualization of TAMs in primary tumors, but also at a distant metastatic site.

**Conclusions:** These data describe, for the first time, a microscopic analysis of (m) and (biv)anti-MMR Nb pharmacokinetics in tumor and healthy tissues. The concepts proposed in this study provide important knowledge for the future use of Nbs as diagnostic and therapeutic agents, especially for the targeting of tumor-infiltrating immune cells.

## Introduction

The development of targeted agents, able to effectively interact with molecular targets involved in tumor progression, has become increasingly important in the management of cancer. These compounds can be conjugated with therapeutic or reporter molecules and can be used for cancer treatment as well as for imaging applications 1, 2. In order to be effective, a proper accumulation of the targeting agent in tumor tissue is required, an aspect that is strongly influenced by the properties of the cancer microenvironment, the physicochemical properties of the targeting agent and the spatial organization of the target molecule. While the pharmacokinetic profile and tumor uptake of targeted agents is generally assessed through chromatography techniques, ELISA, or macroscopic imaging, real-time *in vivo* microscopy to assess the intratumoral accumulation and (sub)cellular localization of compounds is relatively uncommon.

As moieties with a high affinity and specificity, monoclonal antibodies (mAbs) are widely used in targeted tumor therapy and imaging 3, 4. However, their heterotetrameric structure, large size (150kDa) and Fc receptor-mediated recycling limit their penetration in tissue and increase their blood half-life, up to several days or weeks, after systemic administration 5. Although a long circulation time assures high uptake in tumoral tissue, it also causes non-specific accumulation in non-targeted organs and tissues, resulting in unwanted side-effects and a low specificity of imaging. In order to ameliorate the pharmacokinetic and tumor distribution profile, lower molecular weight antibody fragments have been generated 6. Although the smaller dimensions increase their tissue penetration, these molecules are generally prone to denaturation and the formation of immunogenic aggregates, which potentially limit their efficacy 7-9.

An elegant alternative to mAbs (fragments), that solves several of these issues, are Nanobodies (Nbs). Nbs are the variable domain of the homodimeric heavy chain-only antibodies found in camelids and, with an approximate molecular weight of 12-15 kDa, represent the smallest natural antigen binding fragments 10. Compared to conventional monoclonal antibodies and their derived fragments, Nbs are smaller, extremely stable, resistant to aggregation and able to bind their target epitope with nanomolar affinity and reduced steric hindrance. Because of their smaller dimension, Nbs show a higher tissue penetration capability than monoclonal antibodies and a rapid blood clearance, which results in an increased signal to noise ratio in imaging applications 11. From the structural point of view, they show a high similarity with the human type 3 variable heavy chain (VH) domain, but lack the most immunogenic portion of the Fc region, making Nbs only weakly immunogenic in humans 12, 13. Moreover, Nbs can easily be further humanized, thanks to open-source softwares such as Llamanade 14. Nbs are relatively easy to produce and can be easily engineered to generate bivalent Nbs (two identical Nbs connected by a peptide linker), biparatopic Nbs (two distinct Nbs that recognize two epitopes of the same target, connected by a linker) and bispecific Nbs (two distinct Nbs that recognize different antigens, connected by a linker) 14-18. In addition to the use of Nbs as therapeutic drugs in their own right, Nbs have also been conjugated to a broad range of compounds, including imaging probes, drugs, therapeutic radionuclides and nanoparticles 11, 17, 19, 20. Hence, the knowledge of the kinetic properties of different Nb variants and conjugates is crucial to orient the choice toward the most effective diagnostic or therapeutic compound. Indeed, although the pharmacokinetics and the intra-tumoral diffusion of Nbs have been macroscopically described, these aspects remain poorly investigated at the microscopic level *in vivo*.

In the present study, we took advantage of mono- and bivalent anti-Macrophage Mannose Receptor (MMR) Nbs to analyse their kinetics of accumulation, blood retention and cellular targeting *via* non-invasive optical *in vivo* imaging, *ex vivo* confocal microscopy and intravital microscopy 21-23. MMR (CD206) was deemed to be a very suitable target for such a study, since it was previously reported as a marker for hypoxic tumor-associated macrophages (TAMs) that are located deeply within tumor tissue, while also being expressed outside of the tumor tissue 21, 24, 25. We now demonstrate significant differences in the behaviour of monovalent and bivalent anti-MMR Nbs, when administered alone or in combination, both in extra-tumoral and neoplastic tissue. By co-administering labelled monovalent anti-MMR Nbs with an excess of unlabelled bivalent anti-MMR Nbs, these differences can be exploited to improve the visualization or follow-up of TAM infiltration in both primary tumors and distant metastases. In addition, we identified that, in the liver, anti-MMR Nbs target liver sinusoidal endothelial cells (LSEC) instead of liver macrophages, as previously thought. These results provided us with unique insights at a multi-scale level on the pharmacokinetics of Nb-mediated targeting. The increased knowledge obtained in this study is of critical value to orient the choice of the most optimal Nb format for personalized diagnostic or therapeutic interventions in the future.

## Materials and Methods

### Preparation and *in vitro* characterization of fluorescently labelled Nbs

Monovalent monomeric (m), monovalent dimeric (dim) (generated by linking a (m)anti-MMR Nb with an irrelevant BCll10 Nb) and bivalent (biv) constructs of the previously developed anti-MMR Nb (MMRCl1) were produced in bacterial cultures according to standard procedures 21. Briefly, Escherichia coli WK6 cells were transfected with a pHEN6 plasmid coding for the Nb. Nb expression was induced overnight at 28 °C with 1 mM isopropyl-D-thiogalactoside (IPTG). Periplasmic extracts containing the soluble Nbs were obtained by osmotic shock. Nbs were further purified using immobilized metal affinity chromatography on a Ni-NTA resin (Merck) and size-exclusion chromatography (SEC) on a Superdex 75 HR 16/60 column (Cytiva) with PBS as elution buffer. The binding properties of the Nbs were evaluated via Surface Plasmon Resonance (SPR) on a Biacore T200 device (Cytiva) as described in supplementary data 21.

For the subsequent non-invasive imaging and microscopy experiments, anti-MMR Nbs were site-specifically labelled with IRDye680RD (Licor) via a genetically introduced carboxy-terminal cysteine residue 26, or fluorescently labelled via the primary amines in the Nb framework 27 with the Alexa Fluor-647 dye (Invitrogen) or a tri-sulfonated Cy5 dye (Cy5(SO_3_^-^)_2_-SO_3_^-^). The trisulfonated Cy5 dye was synthesized in house as previously described 28, 29. For the site-specific conjugation, Nbs were first reduced using 180-fold molar excess of the mild reductant 2-mercaptoethylamine (2-MEA Hydrochloride, Acros Organics) during 90 min at 37 °C. After buffer exchange to 0.2 M NH4OAc pH 6 using a PD-10 desalting column (GE Healthcare), the reduced Nbs were incubated for 2 h at 37 °C with a 5-fold molar excess of maleimide-derivatized dye. Random labelling of Nbs occurred by reaction with a 5-fold molar excess of NHS-esterified dye (dissolved at 20 mg/mL in DMSO) during 2 h at pH 8.5 (0.1 M K_2_HPO_4_). All reactions were performed in light-protected Eppendorf Tubes. Fluorescent Nbs were subsequently purified by SEC using a Superdex 75 Increase 10/300GL column and PBS (pH 7.4) as elution buffer (0.8 mL/min). The fractions corresponding to fluorescent labelled Nb were collected and pooled. Purity of the final conjugated compounds (>95% purity) and their *in vitro* stability after 3 h at 37 °C in mouse serum (Merck) was confirmed by analytical SEC on a Superdex75 5/150GL column and PBS as running buffer at 0.3 mL/min. The degree of labelling (DOL) was calculated by absorbance measurement (Nanobodrop 2000 spectrophotometer) as the ratio of the fluorophore's to the Nb's concentration. The functionality of the fluorescently labelled Nbs was tested by coating 100 µL of recombinant MMR protein at 2.5 µg/mL in the wells of a Maxisorp 96-well plate (Nunc) for 3 h at room temperature (only PBS was used in the control wells). Following blocking with 200 µL 2% skimmed milk/PBS for 90 min, 100 nM of the fluorescently labelled Nbs (100 µL) was added in triplicate to the wells. After 90 min of incubation at room temperature, the wells were washed three times with PBS + 0.1% Tween and the remaining fluorescent signal was measured via a fluorescent microplate reader (Varioskan LUX) at the appropriate excitation and emission wavelength.

### Animal and tumor model

All mice used were on a C57B/6 background, maintained in a specific-pathogen free facility in individually ventilated cages and given *ad libitum* access to food and water. MMR-deficient (MMR-KO) mice were bred in-house at the Vrije Universiteit Brussel, Belgium. C57Bl/6 wild-type (WT) mice were purchased *via* Charles River Laboratories. Animal experiments were conformed to institutional guidelines, in compliance with national (D.L. N.116, G.U., suppl. 40, 18-2-1992 and N. 26, G.U. March 4, 2014) and international law and policies (EEC Council Directive 2010/63/EU, OJ L 276/33, 22-09-2010; National Institutes of Health Guide for the Care and Use of Laboratory Animals, US National Research Council, 2011). The study was approved by the Italian Ministry of Health (Authorization 158/2011 14/09/2011, ID 6B2B3, approvals n° 207/2016-PR, n° 212/2019-PR and n° 13/2021-PR). All efforts were made to minimize the number of animals used and their suffering.

MN/MCA1 cell line was cultured in RPMI-1640 medium, supplemented with 10% Fetal Bovine Serum (FBS), 1% L-Glutamine, 1% Pen/Strept. In the day of the inoculation, cells were detached with Trypsin/EDTA (Lonza, Basel, Switzerland), washed in saline solution and diluted in saline solution before injection. Subsequently, 50 μL containing 10^5^ MN/MCA1 cells were injected intramuscularly in the mouse hind leg.

### Generation of bone marrow-derived macrophages (BMDMs) and immunofluorescence microscopy

Bone marrow was harvested from the femurs and tibiae of MMR-KO and WT mice and cultured in RPMI 1640 medium supplemented with 10% Fetal Bovine Serum (FBS), 1% L-Glutamine, 1% Pen/Strept. Cells were incubated overnight at 37 °C, 5% CO_2_. After incubation, the cell supernatant was removed and non-adherent cells were collected and cultured on sterile round microscopy glass slides (VWR International) in the same medium and treated with 25 ng/mL of murine M-CSF for 6 days. On day 7, adherent BMDMs were stimulated with murine IFNγ (20 ng/mL) and LPS (100 ng /ml) to induce M1 polarization, or with murine IL-4 (20 ng/mL) to induce M2-polarization. After 24 h, 50 μM Cy5-(m)anti-MMR Nbs were added and cells were incubated for 4 h at 37 °C, 5% CO_2_. Subsequently, cells were washed in PBS with Calcium and Magnesium ions (i.e. PBS^+/+^) and fixed with 4%PFA for 15 min at room temperature, then incubated with primary antibodies diluted in PBS^+/+^ (pH 7.4) containing 2% BSA, 5% normal goat serum, 0.1% Triton X-100 for 1 h at room temperature. Subsequently, cells were washed in PBS^+/+^ (pH 7.4), 0.05% Tween-20 and incubated with Alexa Fluor (488, Cy3)-conjugated secondary antibodies (ThermoFisher) in PBS^+/+^ (pH 7.4) containing 0.05% Tween-20, for 1 h at room temperature. Alternatively, cells were incubated with Alexa Fluor 594-conjugated Phalloidin (ThermoFisher), following the manufacturer's instruction. Cells were then washed with PBS^+/+^ (pH 7.4), 0.05% Tween-20, counterstained with DAPI (ThermoFisher) and mounted with Mowiol. The following antibodies were used: anti-MMR (Abcam, ab64693) and anti-F4/80 (BioRad, MCA497R).

Images were acquired with a Leica TCS SP8 Laser Scanning confocal microscope, equipped with a HC PL APO CS2 63x/1.40 oil or PL APO CS2 100x/1.40 oil objective (Leica), using LASX software. Emission filter bandwidths and sequential scanning acquisition were set up in order to avoid any spectral overlap between fluorophores. Images were then processed using Fiji (ImageJ) software and Imaris (Bitplane) software. If needed, a gaussian filter was applied to representative images shown in the figures to increase their quality.

Fiji (ImageJ) was used to analyse the amount of Cy5-(m)anti-MMR Nbs within polarized macrophages, following the Macro reported in the supplementary information.

### FACS Analysis

Tumors were collected 3 weeks after MN/MCA1 cell injection. Single-cell suspensions were obtained by mechanical and enzymatic dissociation by Type IV Collagenase for 1 h at 37 °C and then resuspended in PBS. Aqua LIVE/Dead Fixable-405 nm (Invitrogen) staining was used to determine cell viability. Subsequently, cells were incubated with Fc block reagent (purified anti-CD16/32, clone 93, eBiosecience/Thermo Fisher Scientific). Then, 50 μL of the antibody mix, diluted in FACS buffer (PBS^-/-^, 2% FCS), was added to each sample. The following antibodies from BD Bioscience or ThermoFisher were used: anti-CD45-BV605 (clone 30-F11), anti-CD11b-BV786 (clone M1/70), anti-F4/80-PeCy7 (clone BM8), anti-Ly6G-PECF594 (clone 1A8), anti-MHCII-PE (clone M5/114.15.2 or 2G9), anti-CD206-APC (clone C068C2). Results are reported as mean fluorescent intensity (MFI) or normalized on isotype control antibody staining. Cells were acquired on LSR Fortessa (BD Bioscience), data collected with DIVA software (v.6.1.1 or 6.2) and analyzed with FlowJo software (Treestar, v.9.9.6).

### Nb circulatory half-life analysis

Tumor-bearing mice were anesthetized with a mixture of ketamine and xylazine and i.v. injected with 2 nmols (based on fluorophore concentration) of IRDye680RD-conjugated (m)anti-MMR Nbs and IRDye680RD-conjugated (biv)anti-MMR Nbs. Blood was collected by tail vein sampling at 2.5, 5, 10, 30 and 60 min after Nb injection using a micropipette (Drummond Scientific Company). Samples were then acquired with the IVIS Lumina III system (Perkin Elmer). Images were analysed using Living Image 4.3.1 software (Perkin Elmer) and data expressed as Radiant Efficiency. Nb circulatory half-life was calculated with GraphPad, using one-phase decay equation.

### *In vivo* fluorescent molecular tomography (FMT)

Fluorescent molecular tomography (FMT) was performed using the FMT2000 system (Perkin Elmer). For 2 weeks before the acquisition, mice were fed with alfalfa-free rodent diet (Mucedola srl or Envigo), in order to reduce autofluorescence background. Mice were anesthetized with a mixture of ketamine and xylazine, shaved to avoid the interference of the fur and i.v. injected with 2 nmoles (based on fluorophore concentration) of IRDye680RD-conjugated (m)anti-MMR Nbs. Where indicated, an excess of unlabelled (biv)anti-MMR Nbs was injected i.p. Mice were positioned in a dedicated imaging cassette that was adjusted to the proper depth to gently restrain the mice. Mice were inserted in dorsal/prone position into the heated docking system of the FMT imaging chamber (37 °C) and trans-illuminated with a 680 nm wavelength near infrared laser. The acquisition took approximately 6-8 min per mouse. Images were collected, reconstructed and analysed using TrueQuant 3.1 software (Perkin Elmer). Three-dimensional regions of interest (ROIs) were designed to select targeted tissues. The total amount of tracer within the ROI was automatically calculated relative to the internal standard generated with a known concentration of IRDye680RD-conjugated (m)anti-MMR Nbs, following the manufacturer's instructions. Although the software provides the results in “pmols” and “nM” of the probe within the ROIs, fluorescence signal can be influenced by a variety of factors related to the propagation of light in tissues (including heterogeneity in tissue density and skin pigmentation): this results in a relative semi-quantification of the signal, which could be better expressed as “Calibrated Fluorescence Signal (CFS)”.

### *Ex vivo* IVIS Lumina III imaging

One hour post Nb administration, mice were sacrificed and perfused with an intracardiac injection of 15-20 mL saline solution. Subsequently, the organs were collected and acquired using the IVIS Lumina III system (Perkin Elmer). Images were analysed using Living Image 4.3.1 software (Perkin Elmer). ROIs were designed in order to select each organ. Data were expressed as Radiant Efficiency.

### Whole Mount tissue analysis

Mice were injected with Nbs, as described in the Results. Subsequently, mice were sacrificed and perfused with an intracardiac injection of 20 mL 2% PFA. Organs were then collected and fixed with 2% PFA overnight at 4 °C. Tissues were incubated with primary antibodies in PBS^+/+^ (pH 7.4), 5% normal goat serum, 0.5% Triton X-100, 0.5%, NaN_3_ for 72 h at 4 °C. Subsequently, tissues were extensively washed using a PBS^+/+^ (pH 7.4), 0.05% Tween-20 solution and incubated with fluorescent secondary antibodies in PBS^+/+^ (pH 7.4), 0.5% Triton X-100, 0.01% NaN_3_ for 48 h at 4 °C. Tissues were then extensively washed using PBS^+/+^ (pH 7.4), 0.05% Tween-20 solution and mounted with Vectashield® mounting medium (Vector Laboratories). The following antibodies were used: Armenian Hamster anti-PECAM-1/CD31 (Merck-Millipore, MAB1398Z), rat anti-mouse F4/80 (BioRad, MCA497R), rabbit anti-Mannose Receptor (Abcam, ab64693), Alexa-Fluor (488, Cy3)-conjugated goat anti-rat (ThermoFisher), Alexa-Fluor (488, Cy3)-conjugated goat anti-rabbit (ThermoFisher), Alexa-Fluor (488, Cy3)-conjugated goat anti-Armenian Hamster (Jackson ImmunoResearch).

Images were acquired as Z-stack with a Leica TCS SP8 Laser Scanning confocal microscope equipped with a HC PL APO CS2 20x/0.75 dry objective or a HC PL APO CS2 40x/1.30 oil objective (Leica). Emission filter bandwidths and sequential scanning acquisition were set up in order to avoid any spectral overlap between fluorophores. Images were then processed using Imaris (Bitplane) software. If needed, a gaussian filter was applied to representative images shown in the figures to increase their quality.

### Intravital Microscopy

#### Liver

Mice were anesthetized with a mixture of ketamine (100 mg/kg) and xylazine (10 mg/kg) and shaved in the abdominal region. The procedure for the exposure and stabilization of the liver was performed as previously described 30. Briefly, a midline laparotomy was performed using scissors to remove the skin. Subsequently, the abdominal muscles were incised using a cauterizer and the liver exposed. Using a knot made by cotton thread, the xiphoid process has been lifted and the falciform ligament between the liver and the diaphragm cut. To visualize liver vasculature, 1 mg of 500 kDa or 2000 kDa MW FITC-conjugated dextran (ThermoFisher) was i.v. injected. In order to inject fluorescent Nbs during the imaging session, a 0.5 mL syringe containing 2 nmoles (based on fluorophore concentration) of fluorescent Nbs was inserted in a fragment of polyethylene tube (PE10) attached to a 30-gauge needle: the needle was then inserted in the retro-orbital sinus. Mice were then placed in the right lateral position on the heated (37 °C) imaging stage (Okolab). Subsequently, the right liver lobe was gently exposed and covered with humid tissues. To maintain proper tissue hydration, a home-made, carbopol-based imaging gel (97% D-Sorbitol, 0.3-0.5% Carbopol 974P, Triethanolamine) was applied to the exposed organ. A piece of tissue soaked with saline solution was used to keep the peritoneal organs (especially the gut) into the abdominal cavity. Liver sections were acquired over time as Z-stack using an Olympus Fluoview FV1000 inverted confocal microscope, equipped with a UPL APO 20X/0.75 dry objective.

#### Healthy and tumor-bearing hind leg

Mice were anesthetized with a mixture of ketamine and xylazine and the left hind leg was shaved. For the visualization of the healthy hind leg, mice were placed in a supine position, while for the imaging of the neoplastic hind leg, mice were placed either in a supine or prone position, based on the shape of the tumor. Using scissors, hind leg skin was carefully removed to expose the underlying muscle or tumor tissue. Tissue was gently cleaned using saline solution. The left hind leg was then firmly blocked using a dedicated intravital imaging stage (provided by the University of Bern, Swiss) and spread with Carbopol-based imaging gel (as previously described). A coverslip was placed on the imaging area and covered with imaging gel. To visualize the vasculature, 1 mg of 2000 kDa MW FITC-conjugated dextran (ThermoFisher) was i.v. injected and 2 nmoles (based on fluorophore concentration) fluorescent Nbs were injected during the imaging session as described before. Tissue sections were acquired over time as Z-stack using a Trimscope II two-photon microscope (LaVision Biotech, Bielefeld, Germany), equipped with a Chameleon Ultra II laser (Coherent) tuned at 820 nm, using a 20X XLUMPlanFLb, NA 1.0 water-immersion objective (Olympus).

### Intravital Microscopy Analysis

Intravital microscopy acquisitions were processed by the Fiji software package (NIH). Where necessary, 3D registration was performed with the “Correct 3D drift” plugin, using the Second Harmonic Generation signal as reference. Acquisitions were then cropped to center the acquired area and Z-projection, using “Sum Slice” as projection type, was applied. As timeframe between stacks, we considered the approximate time taken by the system to acquire an entire Z-stack (4.7 s for the liver and 17.8 s for the healthy and tumor tissue).

To evaluate Nb blood clearance in liver and healthy/tumor tissue, we applied the macro reported in the supplementary information. Briefly, for each acquisition, a threshold was properly set to identify blood vessels, based on the FITC-dextran signal. Subsequently, Nb MFI was calculated over time within the thresholded area. Considering the descending portion of curves relative to the MFI of blood circulating Nbs, the relative fitting curves were computed, to get a function which describes the fluorescence intensity in an analytical form and allows a more precise study of the slope of the fluorescence intensity curves. Subsequently, the first derivatives of the fitting curves were analytically computed and the relative absolute value represented. Higher values of the first derivative correspond to a greater slope and, thus, to a faster kinetic. To analyse Nb macrophage targeting, 3 to 6 square ROIs were designed to select the appearing macrophages. Because of movements due to animal breathing and heart beating, ROI positions were manually adjusted over time to track the selected cells. Then, the MFI within the ROIs was measured. Considering only the ascending portion of the curves relative to the MFI of Nbs, the relative fitting curves were computed, as previously described. Subsequently, the first derivatives of the fitting curves were computed and the relative absolute value plotted.

If needed, a gaussian filter was applied to representative images shown in the figures and movies to increase their quality.

### Statistical Analysis

Statistical analysis was performed using GraphPad Prism software. For IVM analysis, fitting curves were computed using the GraphPad “Growth Curve - Exponential Plateau” toolbox. The Kruskal-Wallis test with Dunn's multiple comparison test and Mann-Whitney test were used as indicated. A p value ≤ 0.05 was considered statistically significant.

## Results

### Far-red and near-infrared dye-labelled anti-MMR Nbs maintain their MMR specificity

To enable *in vivo* and *ex vivo* microscopy, we first labelled monovalent (m) and bivalent (biv) anti-MMR Nbs with either the far-red dyes Cy5 and AF647 or the near-infrared dye IRDye680RD (Figure S1 and Figure S2). For the different fluorescent dyes, a specific conjugation chemistry (amine- and sulfhydryl-reactive chemistry, respectively), previously proven to result in fluorescent Nbs with adequate *in vivo* biodistribution profiles, was chosen 26, 27. The purity and *in vitro* serum stability of the fluorescently labelled monovalent and bivalent Nbs were confirmed *via* size-exclusion chromatography (>95%) (Figure S2A and Figure S2B) and the degree of labelling (DOL) ranged between 0.5 and 0.8. We next ascertained that the labelling did not affect the Nb's binding capacity to MMR *via* an antigen binding assay. This assay demonstrated that fluorescent Nbs bind specifically to wells coated with recombinant MMR antigen and not to uncoated wells (Figure S2C). Additionally, bone marrow-derived monocytes (BMDMs) were isolated from WT and MMR-KO mice, differentiated into macrophages and subsequently polarized with either IFNγ/LPS (M1-polarized) or IL-4 (M2-polarized), conditions that reduce or enhance MMR expression levels, respectively. Polarized macrophages were incubated with Cy5-labelled (m)anti-MMR Nbs for 2 h at 37 °C and analysed by confocal microscopy. As expected, no signal was detected in M1- or M2-polarized macrophages from MMR-KO mice (Figure S2D). Using WT cells, (m)anti-MMR Nbs mainly labelled non-polarized (M0) and M2-polarized macrophages as compared to M1-polarized macrophages (Figure S2E), together suggesting that the fluorescently labelled Nbs maintain their specificity for MMR. This notion was further confirmed by a specific colocalization of (m)anti-MMR Nbs and a conventional anti-MMR monoclonal antibody in M2-polarized macrophages (Figure S2F). These data confirm the specific MMR-targeting capability of fluorescently labelled (m)anti-MMR Nbs *in vitro.*

### Pre-administration of a molar excess of unlabelled bivalent anti-MMR Nbs reduces extratumoral uptake of monovalent anti-MMR Nbs

**Figure 1:**
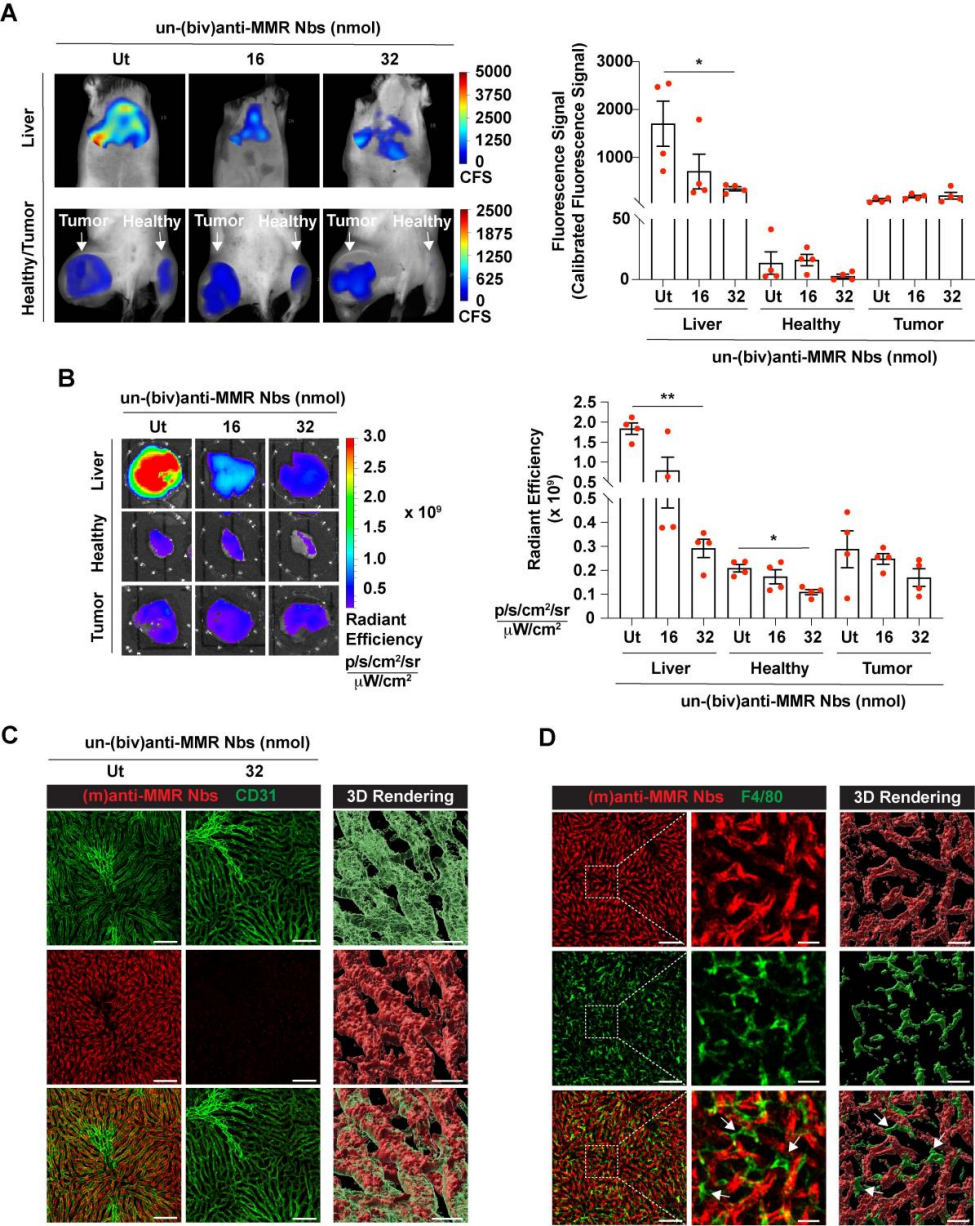
Accumulation of (m)anti-MMR Nbs in liver, healthy and tumor tissue after the administration of a molar excess of unlabelled (biv)anti-MMR Nbs. A) Fluorescent Molecular Tomography analysis of IRDye680RD-labeled (m)anti-MMR Nb accumulation in liver, healthy (muscle) and tumor tissue in 3 weeks tumor-bearing mice upon administration of 0 (Ut), 16 and 32 nmoles of unlabelled (biv)anti-MMR Nbs. Every dot in the graph corresponds to an individual mouse. Data are presented as Mean ± SEM, Kruskal Wallis plus Dunn's Multiple comparison test, * *p<0.05*, ** *p<0.01*. CFS: Calibrated Fluorescence Signal B) *Ex vivo* analysis of IRDye680RD-labeled (m)anti-MMR Nb accumulation in liver, healthy muscle and tumor tissue collected from 3 weeks tumor-bearing mice after administration of 0 (Ut), 16 and 32 nmoles of unlabelled (biv)anti-MMR Nbs. Every dot in the graph corresponds to an individual mouse. Data are presented as Mean ± SEM, Kruskal Wallis plus Dunn's Multiple comparison test, * *p<0.05*, ** *p<0.01*. C) Whole mount confocal microscopy analysis of the colocalization of Cy5-labeled (m)anti-MMR Nbs with CD31, a sinusoidal endothelial cell marker in the liver, with or without the administration of 32 nmoles of unlabelled (biv)anti-MMR Nbs. Scale bar:100 µm. (Close up 3D Rendering Scale Bar: 20 µm. D) Whole mount confocal microscopy analysis in the liver of the localization of Cy5-labelled (m)anti-MMR Nbs and the macrophage marker F4/80. White arrows indicate F4/80^+^ macrophages surrounding liver sinusoidal vessels. Scale bar: 100 µm (Close up 3D rendering Scale bar: 20 µm).

Next, we aimed to visualize MMR^+^ TAMs *in vivo* by optical imaging, using fluorescent molecular tomography (FMT). To this end, C57Bl/6 mice were intramuscularly injected with 10^5^ MN/MCA1 fibrosarcoma cells, a tumor model with a known TAM infiltrate 31. Three weeks after cancer cell injection, tumor infiltration by MMR^+^ TAMs was confirmed by FACS analysis on a tumor single cell suspension (Figure S3). For FMT, mice were shaved, injected i.v. with 2 nmoles of IRDye680RD-labelled (m)anti-MMR Nbs and 1 h later imaged. A higher accumulation of (m)anti-MMR Nbs was observed in the tumor as compared to the corresponding healthy hind leg muscle. However, in line with previous findings, (m)anti-MMR Nbs were also highly retained in the liver (Figure 1A, 1B, and Figure S4A), although the target cell in the liver always remained elusive 21. Confocal analysis on a whole mounted liver lobe now showed that Cy5-labelled (m)anti-MMR Nbs interact with MMR^+^ endothelial cells in liver sinusoids (Figure 1C) and not with the liver macrophages, as previously presumed (Figure 1D).

Before embarking on a more detailed microscopic analysis of (m)anti-MMR Nbs in the tumor microenvironment, we first aimed at confirming a methodology used to reduce the specific extratumoral accumulation of these Nbs. In this respect, we previously demonstrated that the injection of a molar excess of unlabelled (biv)anti-MMR Nbs (un-(biv)anti-MMR Nbs) was able to significantly reduce the on-target, but off-tumor accumulation of tracer amounts of radiolabelled (m)anti-MMR Nbs (especially in the liver), while minimally impacting their tumor targeting potential 21. This observation relies on the assumption that, due to their doubled size and the higher avidity for their target [indeed, due to increased avidity in consequence of the accumulated strength of two binding domains, the bivalent MMR Nbs have a slower dissociation rate compared to the monovalent Nb, resulting in a 5-fold increase in apparent affinity (Figure S1)], (biv)anti-MMR Nbs have a longer clearance and reduced tumor penetration compared to (m)anti-MMR Nbs, while being able to bind to more easily accessible extratumoral MMR^+^ cells and allowing labelled-(m)anti-MMR Nbs to be available to penetrate tumor tissue and target TAMs. To confirm the different clearance rate, we injected i.v. 2 nmoles of IRDye680RD-labelled (m) or (biv)anti-MMR Nbs in 3 weeks tumor-bearing mice, collected blood samples at different timepoints (until 60 min) after Nb injection and analysed the corresponding fluorescent signal. Although both rapidly cleared *via* the kidneys, we found that (biv)anti-MMR Nbs have a longer half-life compared to (m)anti-MMR Nbs [(biv)anti-MMR Nbs : t1/2 10.07 min; (m)anti-MMR Nbs: t1/2 7.03 min], Figure S5A). We then assessed whether the increase in size of (biv)anti-MMR Nbs is hampering their tumor penetration capability compared to (m)anti-MMR Nbs. To this aim, we generated dimeric anti-MMR Nbs [(dim)anti-MMR Nbs] by linking a (m)anti-MMR Nb to an irrelevant BCll10 Nb. The resulting compound has the same avidity for MMR as (m)anti-MMR Nbs (mediated by one MMR-binding site) but a size equal to (biv)anti-MMR Nbs (as a result of the dimeric formulation). Subsequently, 2 nmoles of IRDye680RD-labelled (m), (biv) or (dim)anti-MMR Nbs were injected i.v. in 3-weeks tumor bearing mice. 1 h after Nb injection, mice were sacrificed and tumor and corresponding healthy tissues were imaged. We confirmed that the tumor accumulation of (biv)anti-MMR Nbs was significantly reduced compared to (m)anti-MMR Nbs (Figure S5B). Interestingly, a similar trend was observed for (dim)anti-MMR Nbs (Figure S5B). Of note, the same behaviour was not observed in corresponding healthy tissue, where no differences in the (m), (biv) and (dim)anti-MMR Nb accumulation was detected. These results confirm that, due to differences in size and avidity for the target, (m)anti-MMR Nbs can more easily penetrate dense tumor tissue, while (biv)anti-MMR Nbs can be used as blocking agent for the off-tumor MMR^+^ binding sites, if used at a correct dose.

Indeed, since (biv)anti-MMR Nbs can partially penetrate tumor tissue, their use at a high dose may prevent (m)anti-MMR Nbs from entering the tumor and binding to MMR^+^ TAMs. To reject this assumption, we treated tumor-bearing mice i.p. with an 8x and 16x molar excess of un-(biv)anti-MMR Nbs (16 or 32 nmoles, respectively), 1 h later followed by an i.v. injection of 2 nmoles of IRDye680RD-labelled (m)anti-MMR Nb. *In vivo* (FMT) and *ex vivo* analysis showed a clear reduction in (m)anti-MMR Nb liver accumulation (Figure 1A,1B and Figure S4A) after the administration of 32 nmoles of un-(biv)anti-MMR Nbs. In accordance, confocal microscopy analysis on a whole mount liver lobe demonstrated that the binding of Cy5-labelled (m)anti-MMR Nbs to MMR^+^ liver sinusoids was largely abolished (Figure 1C). A similar reduction in (m)anti-MMR Nb binding was observed in the healthy hind leg muscle (Figure 1A, 1B, and Figure S4A), as well as in other organs, including large intestine, stomach, pancreas, spleen and heart (Figure S6). The competition between (m) and (biv)-MMR Nbs for their binding site was confirmed by analysing the biodistribution of 32 nmoles of AF647-labelled (biv)anti-MMR Nbs upon i.p. injection: *ex vivo* imaging and confocal microscopy showed that, besides the high accumulation in the kidney due to the elimination *via* the urine, (biv)anti-MMR Nbs did accumulate in the liver as well as in healthy tissue (Figure S5C and Figure S5D). Indeed, (biv)anti-MMR Nbs can penetrate also in tumor tissue but, differently from the off-tumor target, the accumulation of (m)anti-MMR Nbs appeared not to be significantly affected by the un-(biv)anti-MMR Nb molar excess administration (Figure 1A, 1B, Figure S5C and Figure S5D).

These data suggest that (biv)anti-MMR and (m)anti-MMR Nbs may bind to TAMs in different tumor regions, due to a different pharmacokinetic behaviour of these compounds.

### Intravital microscopy reveals the kinetics of bivalent and monovalent anti-MMR Nbs in the liver

**Figure 2:**
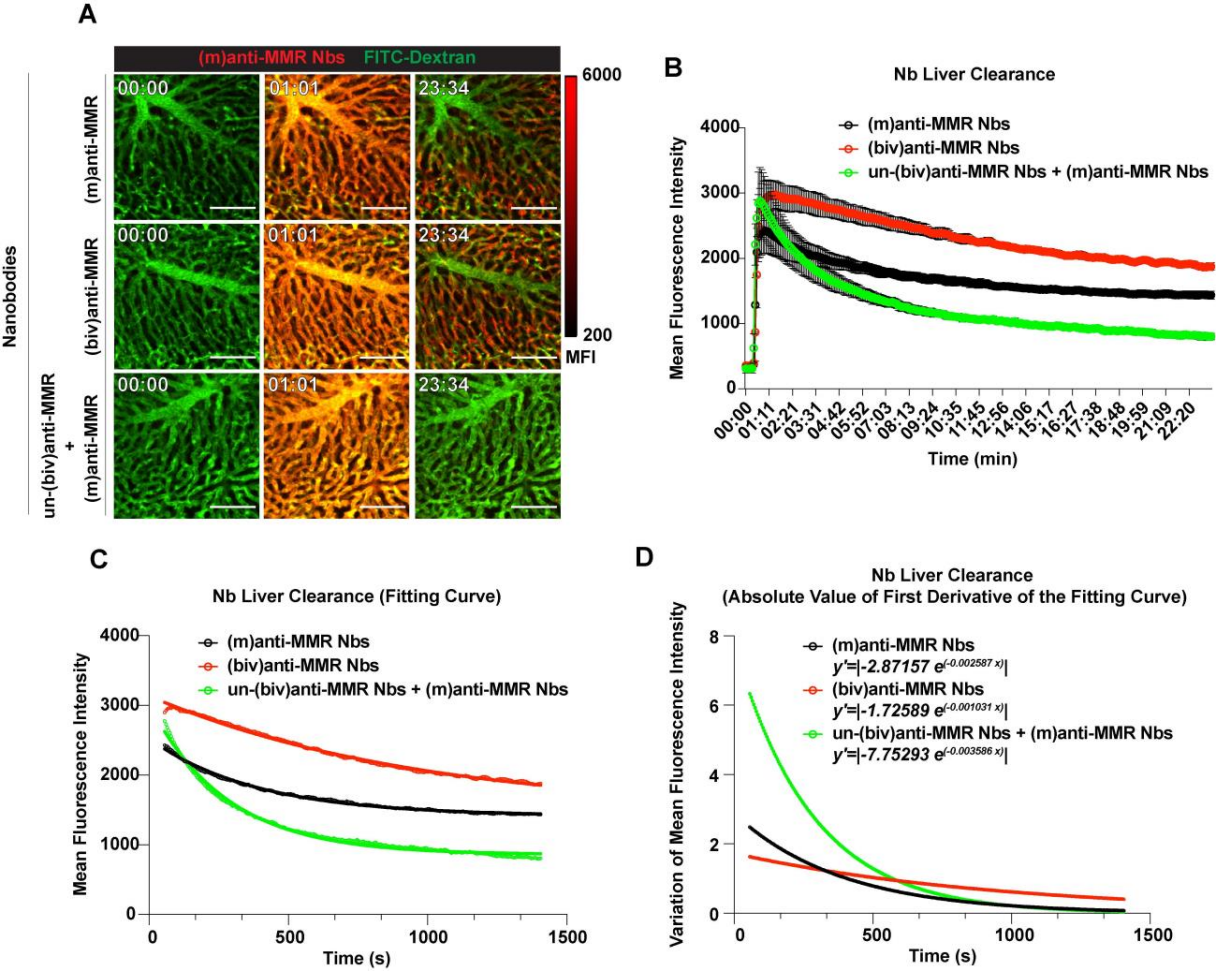
Intravital microscopy (IVM) analysis of (m)anti-MMR Nb and (biv)anti-MMR Nb kinetics in the liver. A) Representative IVM acquisition images of Cy5-labeled (m)anti-MMR Nbs and (biv)anti-MMR Nbs in the liver. Scale bar: 100 µm MFI: Mean Fluorescence Intensity B) Kinetic analysis of (m)anti-MMR Nb and (biv)anti-MMR Nb clearance from the liver vasculature. Mean Fluorescence Intensity of (m)anti-MMR Nbs and (biv)anti-MMR Nbs within liver vessels, in different experimental conditions, was plotted as function of time [(m)anti-MMR Nbs, *n=4*; (biv)anti-MMR Nbs, *n=3*; un-(biv)anti-MMR Nbs + (m)anti-MMR Nbs, *n=3*]. Data are presented as Mean ± SEM. C) Fitting curves computed considering the descending portion of each curve (as shown in panel B), representing the kinetics of Nb clearance from the liver. D) Graphical representation of the absolute value of the first derivative functions analytically computed from the fitting curves shown in panel C.

To obtain a more detailed insight in the kinetics of *in vivo* anti-MMR Nb behaviour, we turned to intravital microscopy (IVM), firstly in the liver. FITC-conjugated dextran was i.v. injected in healthy mice to visualize liver vasculature and, 20 s after the acquisition started, Cy5-labelled anti-MMR Nbs were i.v. injected. When needed, un-(biv)anti-MMR Nbs were injected 1 h before the administration of Cy5-labeled (m)anti-MMR Nbs. Both (m) and (biv)anti-MMR Nbs rapidly reached the liver vasculature and bound to the liver sinusoids within 20 min post injection (Figure 2A top and middle row; Movie S1, Movie S2). In line with previous results, the liver retention of (m)anti-MMR Nbs was strongly impaired by the pre-administration of un-(biv)anti-MMR Nbs (Figure 2A bottom row; Movie S3). To quantify the rate of Nb clearance from the liver in the different experimental conditions [(m)anti-MMR, (biv)anti-MMR, un-(biv)anti-MMR + (m)anti-MMR], the MFI was plotted as function of time (Figure 2B). The descending portion of each curve (from min 00:56 to min 23:25) represents the kinetics of Nb clearance from the liver. Considering this portion of the curves, the relative fitting curves were computed (Figure 2C). Subsequently, the first derivatives of the fitting curves were analytically computed and the relative absolute value represented, then used as a measure of the clearance rate (Figure 2D). (m)anti-MMR Nbs showed a much faster clearance from the liver after the administration of an excess of un-(biv)anti-MMR Nbs (*y'=|-7.75293 e^(-0.003586 x)^|),* compared to either (m)anti-MMR Nbs or (biv)anti-MMR Nbs alone (*y'=|-2.87157 e^(-0.002587 x^)|*and *y'=|-1.72589 e^(-0.001031 x)^|*, respectively). Of note, (biv)anti-MMR Nbs accumulated more readily in the liver compared to (m)anti-MMR Nbs, probably due to their higher affinity for the target (Table 1, Figure 2B and 2C).

Together, these data provide kinetic insights into how un-(biv)anti-MMR Nbs are able to reduce (m)anti-MMR Nb retention in the liver, by blocking the interaction of (m)anti-MMR Nbs with the MMR-expressing liver sinusoid endothelium. Thus, the pre-injection of 32 nmoles of un-(biv)anti-MMR Nbs will be included as a standard treatment in the protocol for the imaging of MMR^+^ TAMs with (m)anti-MMR Nbs.

### Intravital microscopy reveals the blood clearance and macrophage targeting rate of bivalent and monovalent anti-MMR Nbs in the primary tumor microenvironment

**Figure 3:**
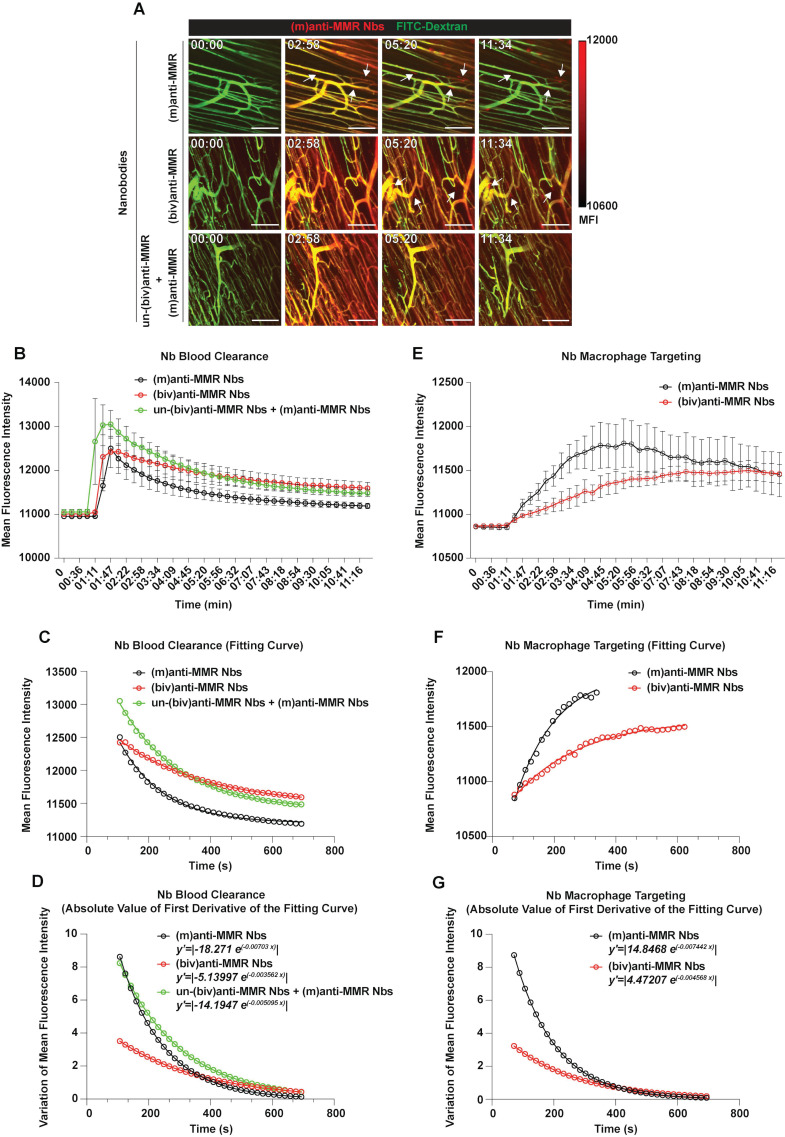
Intravital microscopy (IVM) analysis of the (m)anti-MMR Nb and (biv)anti-MMR Nb macrophage-targeting kinetics in healthy tissue. A) Representative IVM acquisition images of Cy5-labelled (m)anti-MMR Nbs and (biv)anti-MMR Nbs in healthy muscle tissue. White arrows indicate the targeting of tissue-resident macrophages by both (m)anti-MMR Nbs and (biv)anti-MMR Nbs. Scale bar: 100 µm. MFI: Mean Fluorescence Intensity B) Kinetic analysis of (m)anti-MMR Nb and (biv)anti-MMR Nb clearance from healthy muscle tissue vasculature. Mean Fluorescence Intensity of (m)anti-MMR Nbs and (biv)anti-MMR Nbs within healthy tissue blood vessels, in different experimental conditions, was plotted as a function of time [(m)anti-MMR Nbs, *n=3*; (biv)anti-MMR Nbs, *n=4*; un-(biv)anti-MMR Nbs + (m)anti-MMR Nbs, *n=4*]. Data are presented as Mean ± SEM. C) Fitting curves computed considering the descending portion of each curve (as shown in panel B), representing the kinetics of Nb clearance from healthy tissue vasculature. D) Graphical representation of the absolute value of the first derivative functions analytically computed from the fitting curves shown in panel C. E) Kinetic analysis of (m)anti-MMR Nb and (biv)anti-MMR Nb macrophage targeting in healthy muscle tissue. Mean Fluorescence Intensity of (m)anti-MMR Nb and (biv)anti-MMR Nb accumulation on tissue-resident macrophages, in different experimental conditions, was plotted as a function of time [(m)anti-MMR Nbs, *n=3*; (biv)anti-MMR Nbs, *n=4*;]. Data are presented as Mean ± SEM. F) Fitting curves computed considering the ascending portion of each curve (as shown in panel E), representing the kinetics of Nb macrophage targeting in healthy tissue. G) Graphical representation of the absolute value of the first derivative functions analytically computed from the fitting curves shown in panel F.

**Figure 4:**
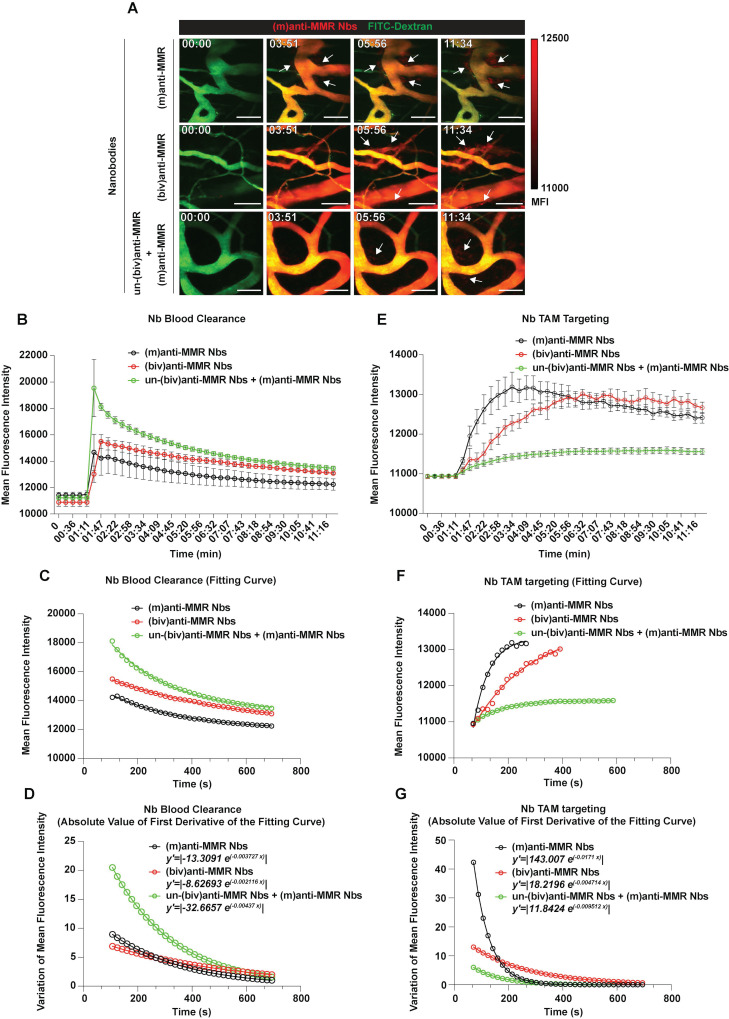
Intravital microscopy (IVM) analysis of the (m)anti-MMR Nb and (biv)anti-MMR Nb macrophage-targeting kinetics in tumors. A) Representative IVM acquisition images of Cy5-labelled (m)anti-MMR Nbs and (biv)anti-MMR Nbs in tumor tissue. White arrows indicate the targeting of TAMs by both (m)anti-MMR Nbs and (biv)anti-MMR Nbs. Scale bar: 100 µm. B) Kinetic analysis of (m)anti-MMR Nb and (biv)anti-MMR Nb clearance from tumor vasculature. Mean Fluorescence Intensity of (m)anti-MMR Nbs and (biv)anti-MMR Nbs within tumor blood vessels, in different experimental conditions, was plotted as a function of time [(m)anti-MMR Nbs, *n=5*; (biv)anti-MMR Nbs, *n=4*; un-(biv)anti-MMR Nbs + (m)anti-MMR Nbs, *n=4*]. Data are presented as Mean ± SEM. C) Fitting curves computed considering the descending portion of each curve (as shown in panel B), representing the kinetics of Nb clearance from tumor vasculature. D) Graphical representation of the absolute value of the first derivative functions analytically computed from the fitting curves shown in panel C. E) Kinetic analysis of (m)anti-MMR Nb and (biv)anti-MMR Nb TAM targeting. Mean Fluorescence Intensity of (m)anti-MMR Nb and (biv)anti-MMR Nb accumulation on TAMs, in different experimental conditions, was plotted as a function of time [(m)anti-MMR Nbs, *n=5*; (biv)anti-MMR Nbs, *n=4*; un(biv)anti-MMR Nbs + (m)anti-MMR Nbs, *n=4*]. Data are presented as Mean ± SEM. F) Fitting curves computed considering the ascending portion of each curve (as shown in panel E), representing the kinetics of Nb TAM targeting. G) Graphical representation of the absolute value of the first derivative functions analytically computed from the fitting curves shown in panel F.

Since pre-treatment with an excess of un-(biv)anti-MMR Nbs strongly lowered (m)anti-MMR Nb retention in the liver, but not in the primary tumor (Figure 1A and 1B), we zoomed in on the kinetics of anti-MMR Nbs in tumor tissue and corresponding healthy muscle tissue by intravital microscopy. To this end, healthy mice or 3 weeks tumor-bearing mice were injected with FITC-dextran to visualize the tissue vasculature and, approximately 20 s after the acquisition started, Cy5-labelled anti-MMR Nbs were i.v. injected [experimental conditions: (m)anti-MMR, (biv)anti-MMR or un-(biv)anti-MMR + (m)anti-MMR, with un-(biv)anti-MMR Nbs injected 1 hour before the administration of Cy5-labelled-(m)anti-MMR Nbs] and the signal was collected over time (Figure 3A and Movie S4, Movie S5 and Movie S6 for healthy tissue; Figure 4A and Movie S7, Movie S8 and Movie S9 for tumor tissue). Following a similar analysis as performed earlier for the liver (considering the descending portion of each curve from min 01:47 to min 11:34) the Nb blood clearance rate was quantified in healthy hind leg muscle and primary tumor tissue (Figure 3B and Figure 4B, Table 1). In healthy tissue, the blood clearance rate of (m)anti-MMR Nbs was not significantly influenced by the pre-administration of un-(biv)anti-MMR Nbs [(m)anti-MMR Nb,* y'=|-18.271 e^(-0.00703 x)^|* and un-(biv)anti-MMR Nbs + (m)anti-MMR Nbs, *y'=|-14.1947 e^(-0.005095 x)^|*] (Figure 3C and 3D, Table 1). Of note, (m)anti-MMR Nbs always showed a faster blood clearance compared to (biv)anti-MMR Nbs (Figure 3C and 3D, Table 1, (biv)anti-MMR Nbs, *y'=|-5.13997 e^(-0.003562 x)^|*). However, the situation in tumor tissue was different, with a (m)anti-MMR Nb blood clearance rate that was significantly increased after the administration of un-(biv)anti-MMR Nbs (Figure 4C and 4D, Table 1, (m)anti-MMR Nbs,* y'=|-13.3091 e^(-0.003727 x)^|*; un-(biv)anti-MMR Nbs + (m)anti-MMR Nbs, *y'=|-32.6657 e^(-0.00437 x)^|*). In addition, the difference in blood clearance rate between (biv)anti-MMR Nbs and (m)anti-MMR Nbs was less pronounced in tumor tissue (Figure 4C and 4D, Table 1, (biv)anti-MMR Nbs, *y'=|-8.62693 e^(-0.002116 x)^|*). These data clearly indicate a different behaviour of blood circulating Nbs in healthy versus tumor tissue.

We then evaluated the kinetics of macrophage targeting by the different Nb formats in healthy and primary tumor tissue. Multiple regions of interest (ROIs) were designed to identify macrophages and, for each ROI, the Mean Fluorescence Intensity (MFI) was measured over time (Figure 3E, 3F and 3G for healthy tissue; Figure 4E, 4F and 4G for tumor tissue; Table 2). Subsequently, the relative fitting curves and the first derivatives of these fitting curves were analytically computed, but only considering the ascending part of the curve in this case, i.e. collecting values from min 01:11 until the peak of Nb accumulation in healthy [(m)anti-MMR Nbs: min 05:38; (biv)anti-MMR Nbs: min 10:23] and tumor tissue [(m)anti-MMR Nbs: min 04:27; (biv)anti-MMR Nbs: min 06:32; un-(biv)anti-MMR Nbs + (m)anti-MMR Nbs: min 09:47].

In healthy tissue, where MMR is mainly expressed by tissue-resident macrophages 32, (m)anti-MMR Nbs targeted macrophages faster than (biv)anti-MMR Nbs (y'=|14.8468 e^(-0.007442 x^)| and y'=|4.47207 e^(-0.004568 x)^|, respectively) (Figure 3F and 3G, Table 2). Moreover, macrophage targeting by (m)anti-MMR Nbs was annihilated by the pre-administration of un-(biv)anti-MMR Nbs, as no signal was detected in the considered time interval (Figure 3A and Movie S6).

Similarly, in tumors, TAM targeting by (m)anti-MMR Nbs was significantly faster compared to (biv)anti-MMR Nbs (Figure 4F and 4G, Table 2, *y'=|143.007 e^(-0.0171 x)^|* and *y'=|18.2196 e^(-0.004714 x)^|*, respectively). Of note, both (m)anti-MMR Nbs and (biv)anti-MMR Nbs showed faster macrophage targeting kinetics in tumor compared to healthy tissue, suggesting an easier Nb diffusion in tumor tissue. Moreover, while in healthy tissue the targeting kinetic of (m)anti-MMR Nbs is approximately 3.3 times faster than (biv)anti-MMR Nbs, this reaches up to 7.8 times faster in tumor tissue, suggesting a more pronounced advantage for (m)anti-MMR Nbs to diffuse in tumor tissue and bind to their target. Importantly, while in healthy tissue no signal from (m)anti-MMR Nbs was detected upon the administration of un-(biv)anti-MMR Nbs, in the tumor the presence of un-(biv)anti-MMR Nbs still allowed (m)anti-MMR Nbs to target TAMs (*y'=|11.8424 e^(-0.009512 x)^|*), albeit with a delayed kinetics and an overall lower fluorescence signal (Figure 4G, Table 2). These data altogether illustrate a different pharmacokinetic of (m) and (biv)anti-MMR Nbs in both healthy and tumor tissue.

### Monovalent anti-MMR Nbs target macrophages at primary and metastatic tumor sites

**Figure 5:**
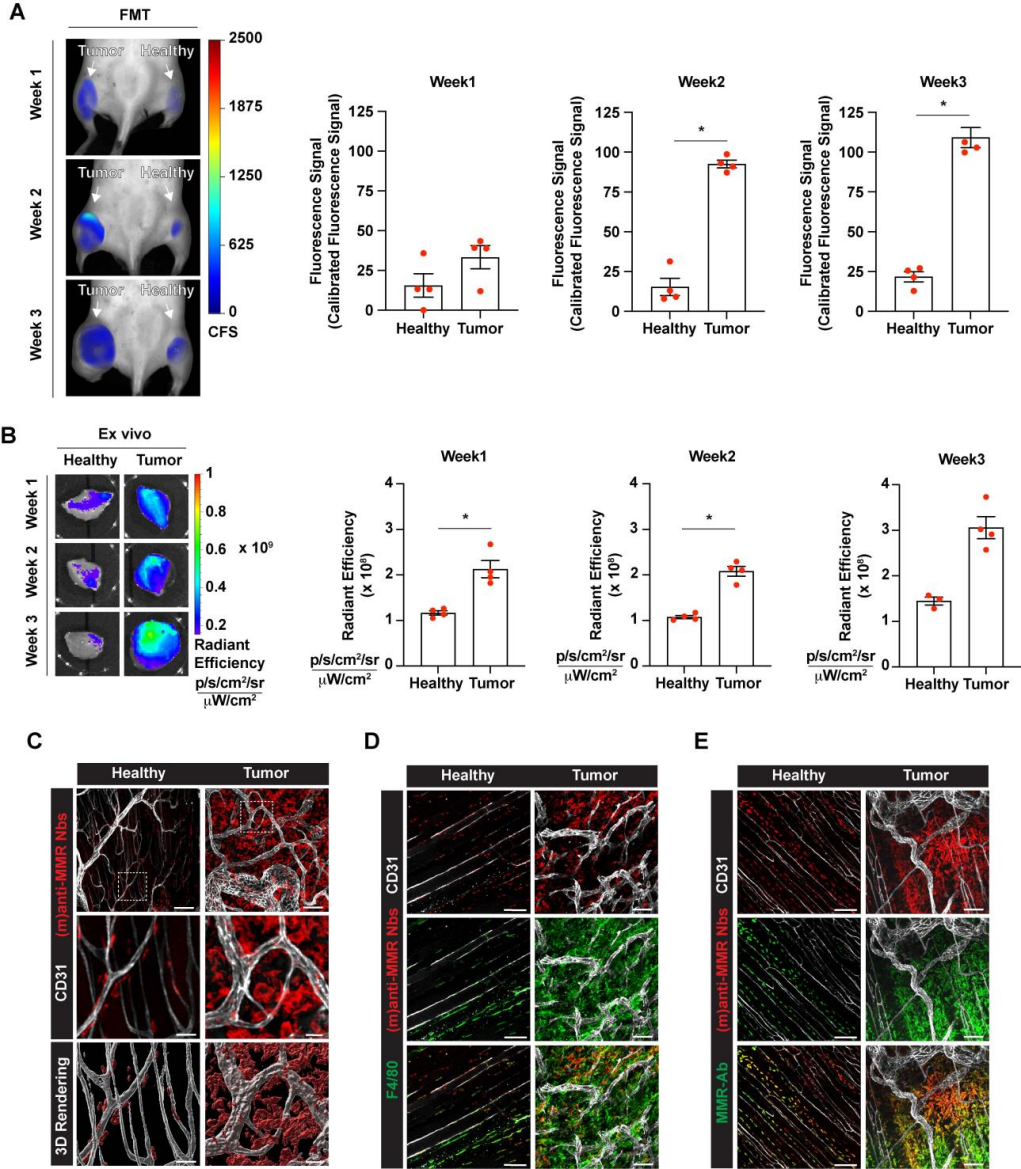
Tumor-associated macrophage targeting by (m)anti-MMR Nbs at the primary tumor site. A) Fluorescent Molecular Tomography of IRDye680RD-labelled (m)anti-MMR Nb accumulation in primary tumor tissue in 1-, 2- and 3-weeks tumor-bearing mice. Every dot in the graph corresponds to an individual mouse. Data are presented as Mean ± SEM. Mann-Whitney test, * p<0.05. CFS: Calibrated Fluorescence Signal B) *Ex vivo* analysis of IRDye680RD-labeled (m)anti-MMR Nb accumulation in healthy muscle and tumor tissue of 1-, 2- and 3-weeks tumor-bearing mice. Every dot in the graph corresponds to an individual mouse. Data are presented as Mean ± SEM. Mann-Whitney test, * p<0.05. C) Whole mount confocal microscopy analysis of Cy5-labelled (m)anti-MMR Nb localization in healthy muscle and tumor tissue. Scale bar:100 µm (Close up scale bar: 20 µm). D) Whole mount confocal microscopy analysis of Cy5-labelled (m)anti-MMR Nb colocalization with F4/80^+^ macrophages. Scale bar healthy: 100 µm; Scale bar tumor: 50 µm. E) Whole mount confocal microscopy analysis of Cy5-labelled (m)anti-MMR Nb colocalization with anti-MMR mAb staining. Scale bar: 100 µm.

Intravital microscopy analyses demonstrated that (m)anti-MMR Nbs and (biv)anti-MMR Nbs have different pharmacokinetic profiles, with (m)anti-MMR Nbs showing an advantage in tumor penetration and TAM targeting. We next evaluated the capability of (m)anti-MMR Nbs to identify MMR^+^ TAMs at different phases of tumor growth. Hereto, 1, 2 and 3-weeks tumor bearing mice were injected with 32 nmoles of un-(biv)anti-MMR Nbs, followed by the administration of 2 nmoles of IRDye680RD-labelled (m)anti-MMR Nbs and then analysed by FMT. A significant accumulation of (m)anti-MMR Nb in the primary tumor was already detected at 1 week post tumor inoculation (when the tumor is still not palpable), with the signal progressively increasing in 2 and 3 weeks tumor-bearing mice (Figure 5A,5B and Figure S4B). Nb retention in the corresponding healthy hind legs was significantly lower at each time point (Figure 5A,5B and Figure S4B). Importantly, confocal microscopic analysis of whole mounted tumor and healthy hind leg demonstrated that Cy5-labelled (m)anti-MMR Nbs specifically bound to F4/80^+^ macrophages in these tissues (Figure 5C and 5D). Of note, not all F4/80^+^ TAMs scored positive for (m)-anti-MMR Nbs, suggesting that these Nbs specifically recognize MMR^+^ TAMs that were not occupied by un-(biv)anti-MMR Nbs. To confirm the MMR-specificity of (m)-anti-MMR Nbs within tumors, we stained the tumor and healthy tissues *ex vivo* with a conventional anti-MMR monoclonal antibody, demonstrating a colocalization of the *in vivo* staining (with (m)-anti-MMR Nbs) and *ex vivo* staining (with anti-MMR mAb) in healthy and neoplastic tissues (Figure 5E).

**Figure 6:**
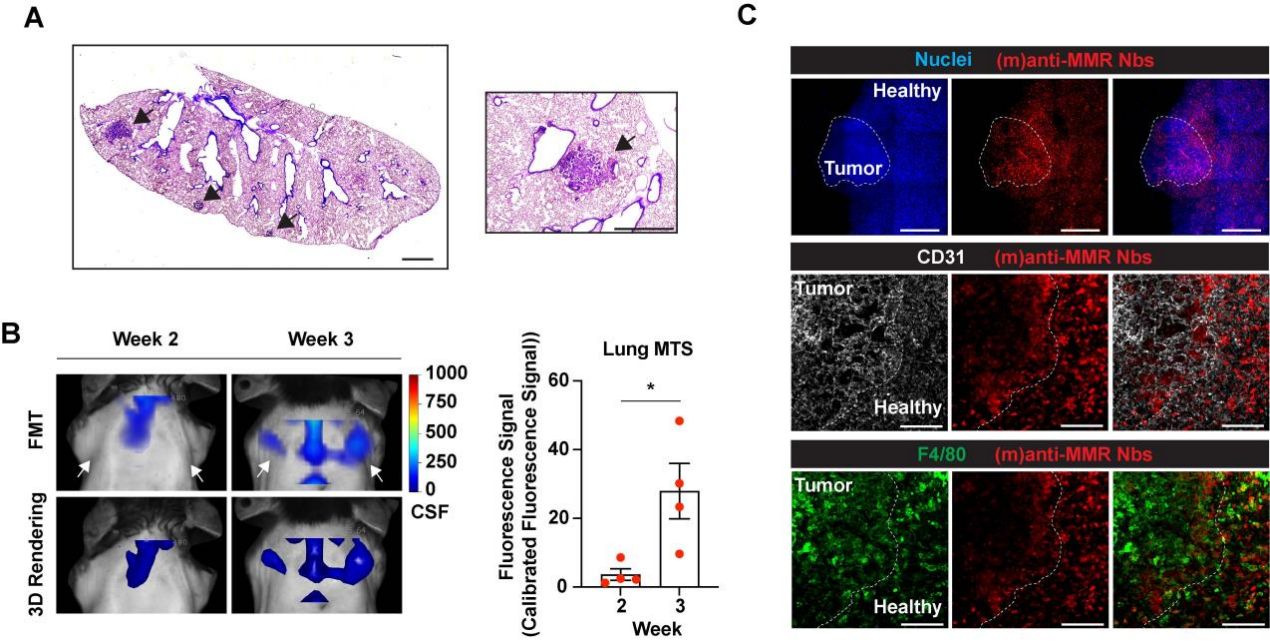
Macrophage targeting by (m)anti-MMR Nbs at the tumor metastatic site. A) H&E staining of the lung of 3-weeks tumor-bearing mice. Black arrows indicate the presence of metastatic lesions. Scale bar: 1mm. B) Fluorescent Molecular Tomography of (m)anti-MMR Nb accumulation at the tumor metastatic site in the lung. White arrows indicate the detection of (m)anti-MMR Nb in the lung metastasis of 3 weeks tumor-bearing mice, compared to 2 weeks tumor-bearing mice. Every dot in the graph corresponds to an individual mouse. Data are presented as Mean ± SEM. Mann-Whitney test, * p<0.05. CFS: Calibrated Fluorescence Signal. C) Whole mount confocal microscopy analysis of (m)anti-MMR Nb accumulation in the lung metastasis of 3 weeks tumor-bearing mice. The white dotted line in the upper panel identifies the presence of a lung metastatic nodule, based on nuclear staining. The yellow dotted line in the middle and lower panels identifies the metastatic front, with (m)anti-MMR Nb colocalizing with F4/80^+^ macrophages. Scale bar: 100 µm

Of note, intramuscularly injected MN/MCA1 fibrosarcoma tumors spontaneously give rise to metastases in the lungs during the third week after cancer cell injection (Figure 6A) 33. Thus, we longitudinally investigated whether IRDye680RD-labelled (m)anti-MMR Nbs were able to detect macrophages not only at the tumor primary site, but also at the lung metastatic site. As for the previous experiments, mice were injected with 32 nmoles of un-(biv)anti-MMR Nbs, followed by the administration of 2 nmoles of IRDye680RD-labelled (m)anti-MMR Nbs. Longitudinal FMT analysis revealed that, at week 3, a specific signal was detected in the region of the lung (Figure 6B and Figure S4C, white arrows), which was absent until week 2, suggesting that (m)anti-MMR Nbs could indeed bind to MMR^+^ macrophages at the developing metastatic site. Confocal microscopic analysis of whole mounted lungs of 3-weeks tumor-bearing mice confirmed the presence of metastases, together with a clear accumulation of Cy5-labelled (m)anti-MMR Nbs (Figure 6C, upper and middle panel). Also in this context, (m)anti-MMR Nbs identified a population of MMR^+^ metastasis-associated macrophages, which seems to preferentially localize at the periphery of the lesion (Figure 6C, lower panel).

These data indicate that (m)anti-MMR Nbs can effectively monitor MMR^+^ macrophage infiltration at different phases of primary tumor growth, but also at the metastatic site.

## Discussion

Nbs are increasingly being considered as innovative and promising tumor targeting agents, based on their small dimensions (~15-20 kDa), high stability/affinity and low immunogenicity 11, 34. We previously generated Nbs able to specifically recognize the expression of MMR on TAMs, for both imaging and therapeutic purposes 21-23, 35. In fact, the imaging of MMR+ TAMs could have a prognostic significance. Indeed, these cells are highly immunosuppressive and pro-angiogenic, so their infiltration is likely to correlate with a worse prognosis and influence the patients' response to immunotherapy 21. Interestingly, ^68^Ga-labeled anti-MMR Nbs recognizing the human homologue of MMR 36 are currently being evaluated in two clinical trials (NCT04168528 and NCT04758650) as tracers for the imaging of MMR+ macrophages via PET/CT in patients with oncological lesions, cardiovascular atherosclerosis, syndrome with abnormal immune activation and cardiac sarcoidosis. Alternatively, anti-MMR Nbs can be envisaged as exquisite vehicles to deliver therapeutic compounds with depleting or reprogramming capacity to this TAM population 23. In the current study, by using a transplanted mouse model of fibrosarcoma, we showed that (m)anti-MMR Nbs accumulate in primary tumor and metastatic lung lesions, specifically targeting MMR^+^ TAMs. The targeting of lung metastasis is of particular interest as lung represents the primary metastatic site of sarcoma and surveillance chest imaging is considered a routine clinical examination for patients with sarcoma 37. Moreover, although pulmonary metastasectomy have been demonstrated to increase patients' survival, an appropriate treatment for metastatic patients has not been established yet 38. In this context, anti-MMR Nbs could be considered a potential useful tool for diagnostic imaging or drug delivery for, respectively, the detection and treatment of sarcoma-derived metastatic lung lesions.

Although the use of radiolabelled anti-MMR Nbs has been macroscopically described, an *ex vivo* and *in vivo* multiscale analysis of monovalent and bivalent anti-MMR Nb pharmacodynamics and real-time kinetics was lacking. In this manuscript, we provide new information about the *in vivo* behaviour of different Nb formats with an unprecedented microscopic detail. Intravital microscopy has previously been successfully applied to investigate the kinetics of diffusion and extravasation of several compounds, including liposomes and nanoparticles 39, 40. Recently, our group applied intravital imaging to study how the size and affinity kinetics of different Nb formulations influence the penetration and the targeting of cancer cells in solid tumors 27. Using a xenograft tumor model in immunodeficient mice, we showed that monovalent anti-HER2 Nbs diffuse more rapidly and homogeneously in tumor tissue, compared to the corresponding bivalent formulation. On the other side, bivalent anti-HER2 Nbs were retained longer in tumor, even if they remained mainly localized in close proximity to the blood vessels.

In the present study, we started from this observation to deepen the investigation on the biodistribution and targeting kinetics of monovalent and bivalent anti-MMR Nbs, at the cellular level, in an orthotopic tumor model in immunocompetent mice. In particular, we did not limit our analysis to tumor tissues, but we explored the behaviour of (m) and (biv)anti-MMR Nbs in healthy tissues and, more interesting, in the liver, which is their on-target, but off-tumor, major accumulation site. In addition, these analyses have been performed injecting (m) and (biv)anti-MMR Nbs alone or in combination, demonstrating how monovalent and bivalent Nbs compete for the binding to their target, both in tumor and off-tumor tissues.

First of all, we demonstrated that, in our experimental conditions, the difference in size and avidity for their target confer to (m) and (biv)anti-MMR Nbs a distinct behaviour, with (biv) Nbs having a longer circulatory half-life and less tumor penetration capability. This observation is noteworthy, since it supports the use of unlabelled (biv)anti-MMR Nbs as blocking agent to saturate the on-target, but off-tumor, MMR binding sites. Importantly, the advantage of (m)anti-MMR Nbs to penetrate neoplastic tissue has been confirmed using (dim)anti-MMR Nbs, which, similarly to (biv)anti-MMR Nbs, accumulate in tumor tissue less than (m)anti-MMR Nbs. Of note, a similar observation has been described with DARPins, which are similar in size to monovalent Nbs and whose dimerization resulted in a significantly lower tumor uptake 41. Thanks to the real time microscopic approach, several important conclusions can be drawn from this study. Upon i.v. injection, (m)anti-MMR Nbs more readily accumulate in tumor tissue compared to corresponding healthy tissues. However, a high liver retention was observed, due to the specific interaction of (m)anti-MMR Nbs with MMR^+^ CD31^+^ liver sinusoidal endothelial cells (LSEC), but not with liver macrophages. The latter is remarkable, considering the fact that MMR is best known as a macrophage marker and is likely explained by the higher accessibility of LSEC. Intravital microscopy on the liver clearly demonstrated that, due to their higher avidity, the injection of a molar excess of un-(biv)anti-MMR Nbs mostly prevented (m)anti-MMR Nb interaction with LSEC, thus strongly increasing their clearance from the liver and their bioavailability. Similarly, un-(biv)anti-MMR Nb administration also influenced the (m)anti-MMR Nb kinetics in healthy tissue. Although the (m)anti-MMR Nb blood clearance was not significantly modified, their interaction with tissue macrophages was impaired, as no targeting was detected in the considered time interval. This prevention of extra-tumoral binding of (m)anti-MMR Nbs is especially crucial to avoid therapy-induced side toxicity when anti-MMR Nb-drug conjugates are used, for example in the setting of MMR Nb-based radioimmunotherapy 22, 42, 43. In fact, the occurrence of an antigenic sink, caused by the expression of the molecular target on various cells in the body (besides the target cell), is a major concern for antibody/Nb-mediated therapies and has, therefore, a great clinical significance. Indeed, antigenic sinks are responsible for toxicities and for diminished effectivity of the therapeutic antibody/Nb, so several strategies to reduce these effects are currently investigated 44, 45. The use of (biv)anti-MMR Nbs, as we proposed, clearly leads to a reduction of the peripheral antigenic sink. Of note, a similar strategy has been previously applied for the antibody-mediated blockade of several therapeutic targets, such as CD20, CD47 and Neuropilin-1 46-48. In addition, the pre-administration of an excess of unlabelled tracer to block constitutively expressed target molecules is a common strategy in the context of fluorescence-guided surgery. For example, in patients, it has been demonstrated that a loading dose of 100 mg of unlabelled cetuximab or panitumumab saturates EGFR expression in normal tissue and, thus, optimizes tumor-to-background contrast subsequently obtained with fluorescently labelled cetuximab or panitumumab 49-51.

Whole mount confocal analysis and intravital microscopy experiments showed that (m)anti-MMR Nbs and (biv)anti-MMR Nbs do reach the tumor tissue and do bind to TAMs. Interestingly, FMT and *ex vivo* imaging data demonstrated that the administration of un-(biv)anti-MMR Nbs does not significantly impact on the overall accumulation of (m)anti-MMR Nbs in the tumor, contrary to healthy tissues. This difference may be due to the higher MMR^+^ macrophage infiltration in tumor compared to healthy tissue, resulting in an incomplete occupancy of these cells by the un-(biv)anti-MMR Nbs. In addition, the tissue architecture and microenvironmental cues within tumors usually differ from the corresponding healthy tissues, allowing a different spatial distribution of MMR^+^ macrophages. For example, hypoxic areas are more typically found within tumors and are known to accumulate macrophages, which may be more easily reached by (m)anti-MMR Nbs, but not (biv)anti-MMR Nbs. As a matter of fact, the presence of (biv)anti-MMR Nbs in tumor tissue creates binding competition. IVM showed that the pre-treatment with un-(biv)anti-MMR Nbs lowers the TAM targeting kinetic of (m)anti-MMR Nbs, however still allowing the latter to efficiently reach TAMs and to generate a high detectable signal. This observation suggests that, due to their differences in tissue diffusion capacity and avidity for the target, (biv)anti-MMR Nbs and (m)anti-MMR Nbs occupy different MMR+ TAM populations, with (biv)anti-MMR Nbs targeting the perivascular tumor regions and likely forcing the (m)anti-MMR Nbs to diffuse further into the tumor tissue and bind MMR+ TAMs in less accessible regions. These aspects are, depending on the type of drug to be delivered, likely to be decisive for achieving an effective therapeutic response. Hence, single therapies with a direct and very local apoptotic effect (e.g. alpha-radiation) will necessitate delivery deep into the tumor, while perivascular delivery can be sufficient for therapeutic strategies triggering a cascade of reactions through the bystander effect (beta-radiation therapy, photodynamic therapy, etc).

Finally, it cannot be excluded that TAMs display a higher phagocytic activity compared to other macrophages, which could result in a higher MMR recycling rate and a faster internalization of MMR/un-(biv)anti-MMR Nb complexes. This may lead to the surface exposure of new MMR molecules, which can in turn be targeted by (m)anti-MMR Nbs.

Another important observation is that (m)anti-MMR Nbs showed a faster blood clearance and a higher blood availability in tumor tissue, but remarkably not in healthy tissue, when injected after the administration of un-(biv)anti-MMR Nbs. The faster blood clearance in tumors could be linked to the fact that neoplastic growth and overexpression of pro-angiogenic factors result in the development of a disorganized network of immature and hyper-permeable blood vessels 52, 53. The effectiveness of drug delivery to tumors is influenced by a variety of factors, including the enhanced permeability and retention (EPR) effect, the plasma pharmacokinetics, as well as the properties and plasma concentration of the drug 53-55. It is assumed that, as a result of the blocking strategy, especially in the liver, the bioavailability and plasma concentration of (m)anti-MMR Nbs increase. Indeed, IVM analysis showed a higher (m)anti-MMR Nb vascular signal, immediately after injection, in mice treated with un-(biv)anti-MMR Nbs. Notably, this enhancement is more evident in tumor than in healthy tissue. Although the kinetics of drug diffusion and accumulation in tumors are extremely complex, we can speculate that the administration of a molar excess of un-(biv)anti-MMR Nbs could increase the plasma concentration of (m)anti-MMR Nb. Due to the abnormal structure and density of the neoplastic vasculature, this effect is particularly pronounced in tumor tissue, leading to a faster extravasation of (m)anti-MMR Nbs through the hyper-permeable vascular endothelium.

Finally, it is important to highlight that (m)anti-MMR Nbs and (biv)anti-MMR Nbs behave differently in both healthy and tumor tissues. With regards to the blood clearance rate, we found that, in healthy tissue, (m)anti-MMR Nbs showed a faster kinetic than (biv)anti-MMR Nbs. Interestingly, such a difference has not been observed anymore in neoplastic tissue, which is in line with the increased tumor vascular permeability and blood leakage. Indeed, while in healthy tissue the diffusion of (m)anti-MMR Nbs through the endothelium is facilitated by their smaller dimension, in tumor, where the blood vessel architecture is significantly altered, the difference in size between (m)anti-MMR Nbs and (biv)anti-MMR Nbs is less determinant and the kinetics of diffusion through the vessel wall is comparable. Similarly, the difference in size between (m)anti-MMR Nbs and (biv)anti-MMR Nbs influences their targeting capability: in fact, both in healthy and tumor tissue, (m)anti-MMR Nbs showed a faster kinetic of macrophage targeting compared to (biv)anti-MMR Nbs. More interestingly, both (m)anti-MMR Nbs and (biv)anti-MMR Nbs showed a faster macrophage targeting kinetic in tumors compared to healthy tissue, suggesting an easier Nb diffusion in tumor tissue. In particular, the advantage of (m)anti-MMR Nbs to interact with their target more rapidly, as observed in healthy tissue, is even more pronounced in tumor tissue. This result strongly confirms that, due to their smaller dimension and lower avidity, (m)anti-MMR Nbs can diffuse and penetrate easier into tumor tissue compared to the larger and more avid (biv)anti-MMR Nbs.

## Conclusions

Together, our *ex vivo* and *in vivo* microscopic data provide an unprecedented kinetic insight in the behaviour of monovalent versus bivalent Nbs that target a tumor-infiltrating stromal cell, i.e. tumor-associated macrophages. An important conclusion lies in the observation that both monovalent and bivalent Nbs target macrophages more readily in tumor tissue as compared to normal tissue, but this enhanced diffusion within tumors is clearly more pronounced for monovalent Nbs, illustrating their selective advantage for tumor targeting. A major hurdle for any Nb- or mAb-based tumor targeting is the existence of a peripheral antigenic sink. We could demonstrate that a pre-treatment with unlabelled bivalent Nbs not only occupies extratumoral binding sites, but also leads to a higher blood availability of the labelled monovalent Nb in tumor tissue. Hence, bivalent Nbs have the capacity to prevent the antigenic sink, while still allowing the monovalent Nbs to enter the tumor and bind their target molecule.

Altogether, these data provide clinically relevant information on the different behaviour of (m) and (biv)anti-MMR Nbs, which will be used to orient the ongoing phase I/II studies we are leading on the application of ^68^Ga-labeled (m)anti-MMR Nb as a new tracer for PET/CT imaging in patients with oncologic lesions (included head & neck cancer, breast cancer and lung cancer) or cardiovascular atherosclerosis (NCT04168528, NCT04758650).

Moreover, the concept proposed in this study will be translatable to other Nbs directed against other markers expressed on tumor-infiltrating immune cells, thus providing important knowledge for future Nb-based cancer therapies.

## Supplementary Material

Supplementary figures, movie legends, materials and methods.

Movie S1.

Movie S2.

Movie S3.

Movie S4.

Movie S5.

Movie S6.

Movie S7.

Movie S8.

Movie S9.

## Figures and Tables

**Table 1:** Pharmacokinetic of Nb clearance

Nb combination	Liver	Healthy tissue	Tumor
**(m)anti-MMR Nbs**	*y'=|-2.87157 e^(-0.002587 x)^|*	*y'=|-18.271 e^(-0.00703 x)^|*	*y'=|-13.3091 e^(-0.003727 x)^|*
**(biv)anti-MMR Nbs**	*y'=|-1.72589 e^(-0.001031 x)^|*	*y'=|-5.13997 e^(-0.003562 x)^|*	*y'=|-8.62693 e^(-0.002116 x)^|*
**un-(biv)anti-MMR Nbs + (m)anti-MMR Nbs**	*y'=|-7.75293 e^(-0.003586 x)^|*	*y'=|-14.1947 e^(-0.005095 x)^|*	*y'=|-32.6657 e^(-0.00437 x)^|*

**Table 2:** Pharmacokinetic of Nb macrophage targeting in healthy tissue and tumor (*ND: not detectable*)

Nb combination	Healthy tissue	Tumor
**(m)anti-MMR Nbs**	*y'=|14.8468 e^(-0.007442 x^)|*	*y'=|143.007 e^(-0.0171 x)^|*
**(biv)anti-MMR Nbs**	*y'=|4.47207 e^(-0.004568 x)^|*	*y'=|18.2196 e^(-0.004714 x)^|*
**un-(biv)anti-MMR Nbs + (m)anti-MMR Nbs**	*N.D.*	*y'=|11.8424 e^(-0.009512 x)^|*

## Abbreviations

Nbs: Nanobodies
MMR: Macrophage Mannose Receptor
LPS: Lipopolysaccharide
IL-4: Interleukin-4
IFNg: Interferon-g
TAMs: Tumor-Associated Macrophages
