# Supplementary material for: Size-advantage of monovalent nanobodies against the macrophage mannose receptor for deep tumor penetration and tumor-associated macrophage targeting
Source: Theranostics. 2023 Jan 1;13(1):355–73. doi: 10.7150/thno.77560 (PMC9800720; doi:10.7150/thno.77560)
Supplement: Supplementary file 1 — Supplementary figures, movie legends, materials and methods. [file thnov13p0355s1.pdf]

Supporting information:

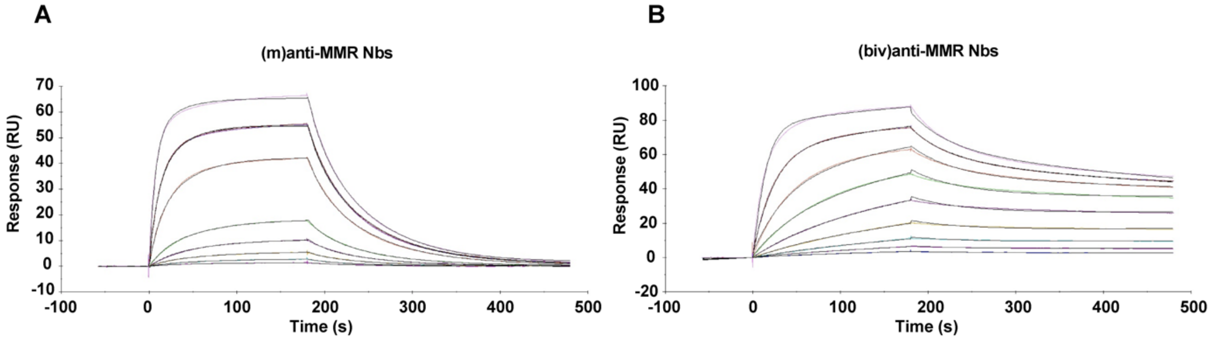

	Ka1 (1/Ms)	Kd1 (1/s)	Ka2 (1/s)	Kd2 (1/s)	KD (M)	Chi² (RU²)
(m)anti-MMR Nb	9.820E+5	0.04196	0.01746	0.02463	2.500E-8	0.0607
(biv)anti-MMR Nb	8.563E+5	0.01680	0.006332	0.002158	4.987E-9	0.267
(dim)anti-MMR Nb	4.229E+5	0.03470	0.01196	0.02729	5.706E-8	0.0138

C

(m)anti-MMR Nb sequence with His tag

QVQLQESGGGLVQPGGSLRLSCAASGNIFSINAIGWYRQAPGKQRELVATITLSGSTNYA
DSVKGRFSISRDNAKNTVYLQMNSLKPEDTAVYYCNANTYSDSDVYGYWGQGTQVTVSSHHHHHH

(biv)anti-MMR Nb sequence with His tag

QVQLQESGGGLVQPGGSLRLSCAASGNIFSINAIGWYRQAPGKQRELVATITLSGSTNYA
DSVKGRFSISRDNAKNTVYLQMNSLKPEDTAVYYCNANTYSDSDVYGYWGQGTQVTVSSG
GGGSGGGGSGGGGSQVQLQESGGGLVQPGGSLRLSCAASGNIFSINAIGWYRQAPGKQRE
LVATITLSGSTNYADSVKGRFSISRDNAKNTVYLQMNSLKPEDTAVYYCNANTYSDSDVY
GYWGQGTQVTVSSHHHHHH

(dim)anti-MMR Nb sequence with His tag

QVQLQESGGGLVQPGGSLRLSCAASGNIFSINAIGWYRQAPGKQRELVATITLSGSTNYADSVKGRFSISRDNAKNTVYL
QMNSLKPEDTAVYYCNANTYSDSDVYGYWGQGTQVTVSSAHHSEDPSSKAPKAPMAQVQLVESGGGSV
QA GGSLRLSCTASGGSEYSYSTFSLGWFRQAPGQEREAVAAIASMGGLTYYASVKGRFTISRDNAKNTVTL
QMNNLKPEDTAIYYCAAVRGYFMRLPSSHNFRYWGQGTQVTVSSHHHHHH

D

Molecular Weight of different (m), (biv) and (dim) constructs

	MMRCl1 (monovalent)	MMRCl1-MMRCl1 (bivalent)	MMRCl1-BCII10 (dimeric)
**No Tag**	*12801 Da*	*28354 Da*	*26529 Da*
**6His tag**	*13625 Da*	*29178 Da*	*27353 Da*
**Cys**	*15059 Da*	*30612 Da*	*28787 Da*

Supplementary Figure 1. Sequence and binding kinetics of the (m)anti-MMR Nb, (biv)anti-MMR Nb and (dim)MMR Nb on recombinant mouse MMR protein. (A) Association and

dissociation kinetic profiles of anti-MMR monovalent and bivalent Nbs as determined *via* surface plasmon resonance (SPR). (B) Calculated binding kinetic parameters k_a: association rate, k_d: dissociation rate, K_D: dissociation constant for (m)anti-MMR Nb, (biv)anti-MMR Nb and (dim)MMR Nb. C) Amino acid sequence coding for the (m)anti-MMR Nb, (biv)anti-MMR Nb and (dim)anti-MMR Nb containing a C-terminal hexahistidine tag. D) Molecular weight of different (m), (biv) and (dim) constructs.

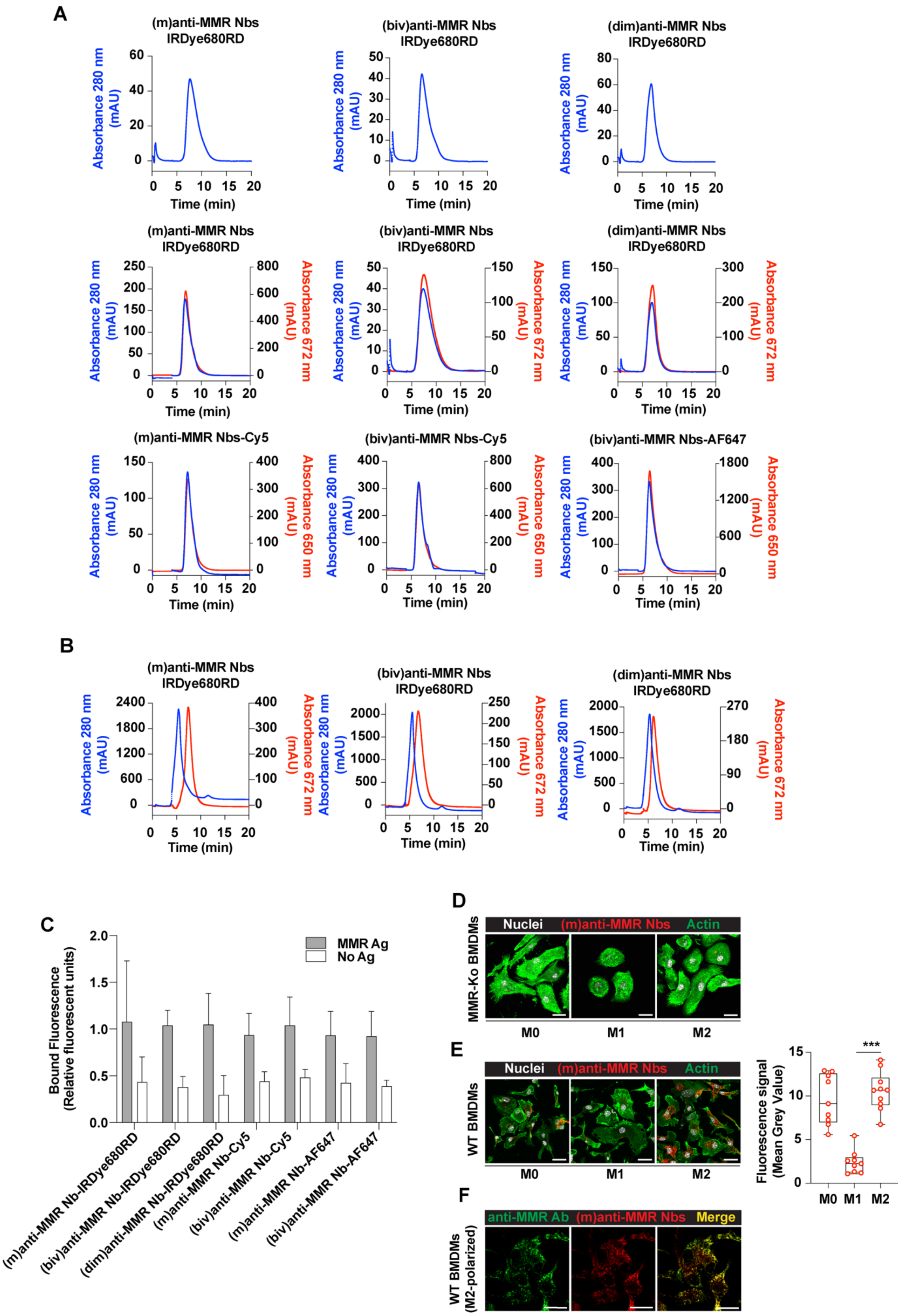

A
(m)anti-MMR Nbs IRDye680RD
(biv)anti-MMR Nbs IRDye680RD
(dim)anti-MMR Nbs IRDye680RD
Absorbance 280 nm (mAU)
Absorbance 672 nm (mAU)
Time (min)
(m)anti-MMR Nbs-Cy5
(biv)anti-MMR Nbs-Cy5
(biv)anti-MMR Nbs-AF647
Absorbance 650 nm (mAU)
B
C
Bound Fluorescence (Relative fluorescent units)
MMR Ag
No Ag
(m)anti-MMR Nb-IRDye680RD
(biv)anti-MMR Nb-IRDye680RD
(dim)anti-MMR Nb-IRDye680RD
(m)anti-MMR Nb-Cy5
(biv)anti-MMR Nb-Cy5
(m)anti-MMR Nb-AF647
(biv)anti-MMR Nb-AF647
D
MMR-Ko BMDMs
Nuclei (m)anti-MMR Nbs Actin
M0 M1 M2
E
WT BMDMs
Fluorescence signal (Mean Grey Value)
***
F
WT BMDMs (M2-polarized)
anti-MMR Ab (m)anti-MMR Nbs Merge

Supplementary Figure 2. Generation and validation of far-red and near infra-red dye-labelled anti-MMR Nbs. A) Validation of the purity of unlabelled, far-red and near infra-red dye-labelled anti-MMR Nbs by size exclusion chromatography. B) Demonstration of the *in vitro* stability of fluorescently labelled anti-MMR Nbs following incubation in serum for 3 h at 37 °C by size exclusion chromatography. C) Binding assay on recombinant MMR protein demonstrating the functionality of the fluorescently labelled MMR Nbs. D) Immunofluorescence confocal microscopy analysis of polarized bone marrow-derived macrophages, isolated from MMR-KO mice and incubated with Cy5-labelled (m)anti-MMR Nbs. Scale bar: 20 μm. E) Immunofluorescence confocal microscopy analysis of polarized bone marrow-derived macrophages, isolated from WT mice and incubated with Cy5-labelled (m)anti-MMR Nbs. Scale bar: 20 μm. Data are presented as box and whiskers plot (Min to Max). Every dot in the graph corresponds to a different image. Mann-Whitney test, *** $p<0.001$. F) Immunofluorescence confocal microscopy analysis of M2-polarized macrophages, isolated from WT mice, incubated with Cy5-labelled (m)anti-MMR Nbs and co-stained with a monoclonal anti-MMR antibody revealed by AF488-labelled secondary antibody. Scale bar: 20 μm.

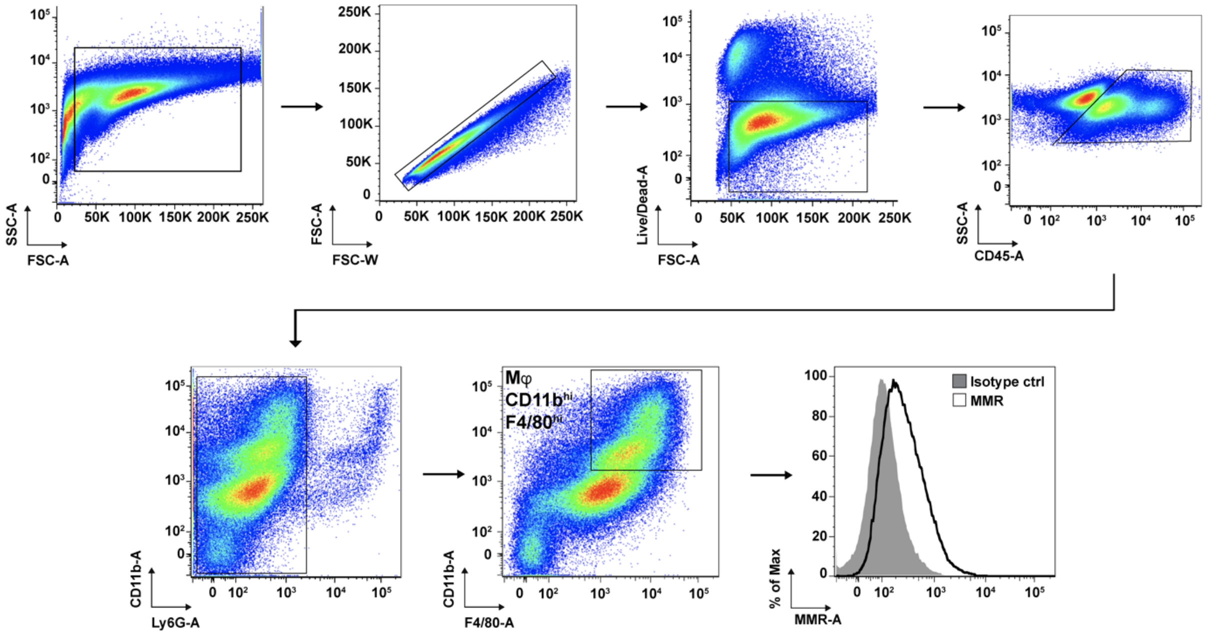

Supplementary Figure 3. FACS analysis of MMR expression on tumor-associated macrophages. Gating strategy to evaluate the expression of MMR by tumor-infiltrating macrophages is shown.

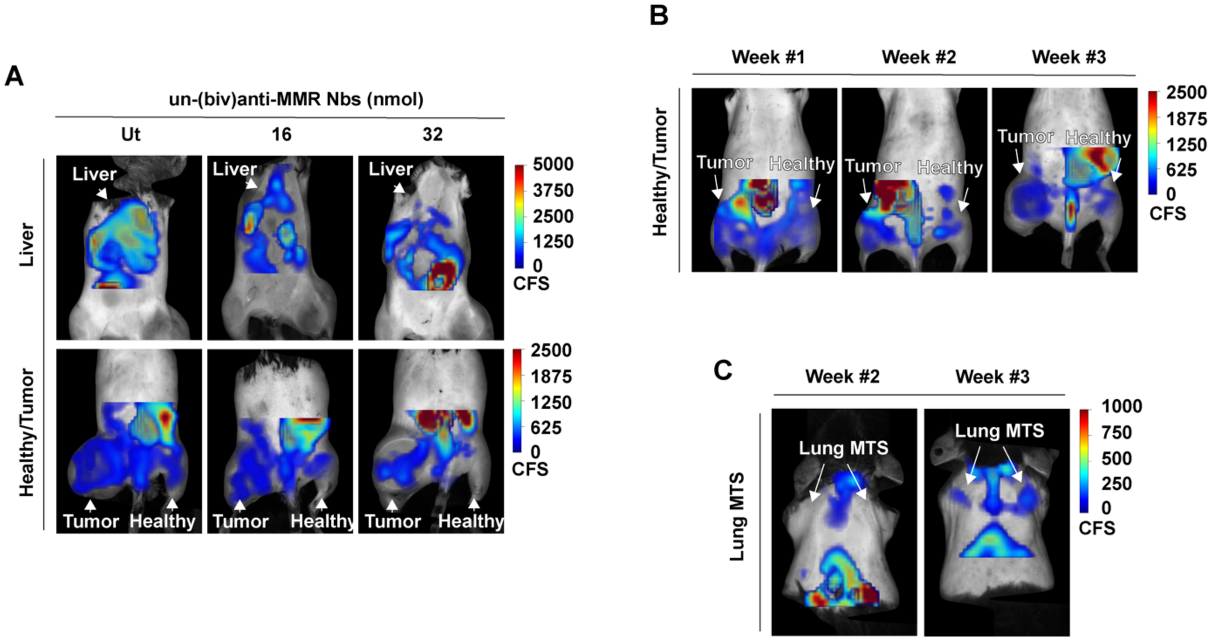

Supplementary Figure 4: Raw FMT fluorescence signal detected in tumor-bearing mice 1 hour after the injection of IRDye680RD-conjugated (m)anti-MMR Nbs. A) IRDye680RD-conjugated (m)anti-MMR Nbs accumulation in the abdominal cavity and tumor/healthy leg region, with or without the pre-administration of different doses of unlabelled (biv)anti-MMR Nbs. White arrows indicate the signal coming from the liver (upper panel) and primary tumor/corresponding healthy tissue (lower panel). CFS: Calibrated Fluorescence Signal. B) IRDye680RD-labeled (m)anti-MMR Nb accumulation in primary tumor/healthy leg region in 1-, 2- and 3-weeks tumor bearing mice. White arrows indicate the signal coming from the primary tumor/corresponding healthy tissue. CFS: Calibrated Fluorescence Signal. C) IRDye680RD-labeled (m)anti-MMR Nb accumulation in thoracic cavity in 2- and 3-weeks tumor bearing mice. White arrows indicate the occurrence of lung metastases 3 weeks after tumor cell injection. CFS: Calibrated Fluorescence Signal.

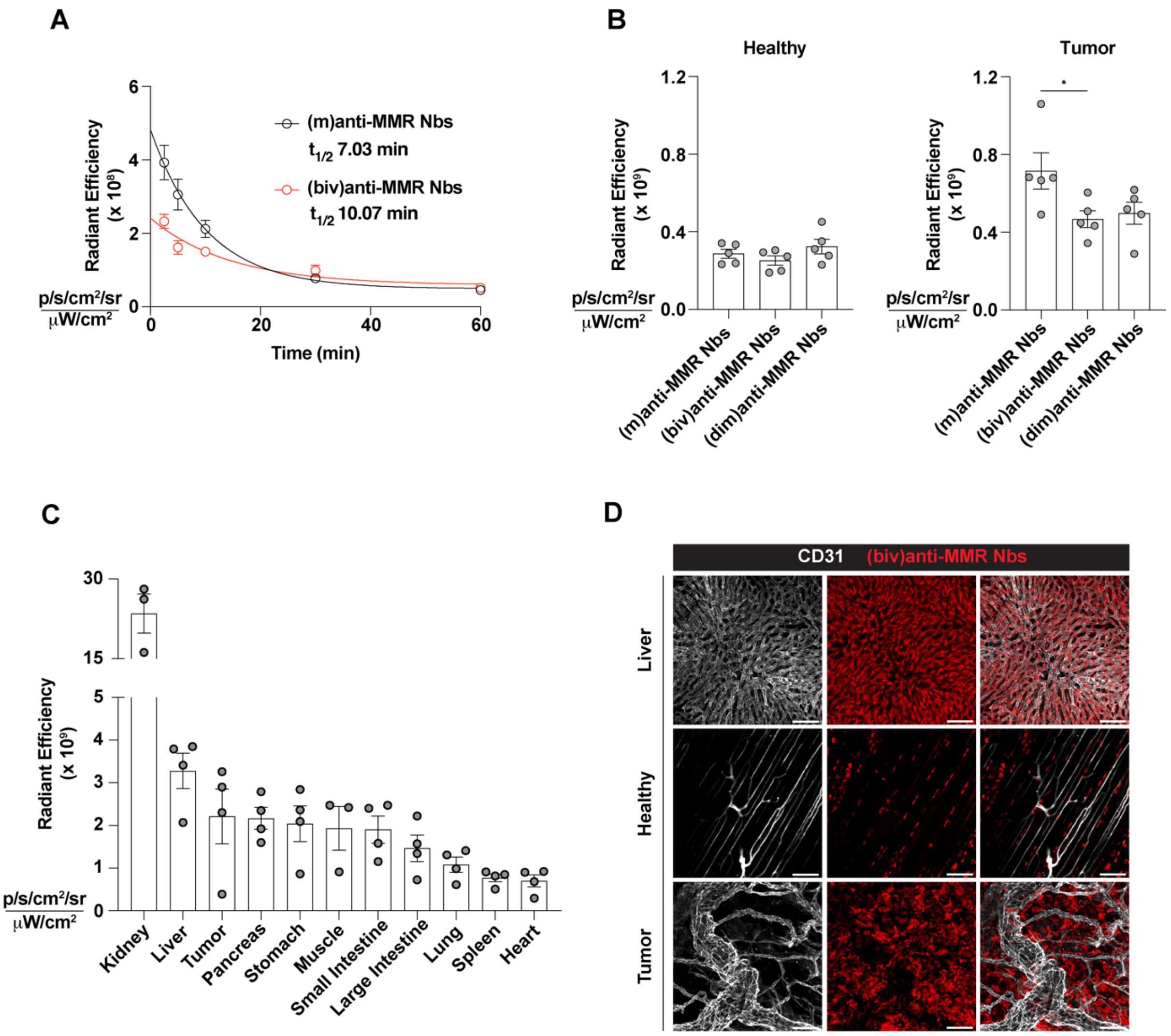

Supplementary Figure 5. A) Circulatory half-life of 2 nmoles of IRDye680RD-conjugated (m) and (biv)anti-MMR Nbs. Data are presented as Mean ± SEM. One phase decay, *n=5* B) Penetration of 2 nmoles of IRDye680RD-conjugated (m), (biv) and (dim)anti-MMR Nbs in tumor and corresponding healthy tissue. Every dot in the graph corresponds to an individual mouse. Data are presented as Mean ± SEM. Kruskal-Wallis plus Dunn's Multiple comparison test, * $p<0.05$. C) *Ex vivo* biodistribution analysis of 32 nmoles of AF647-labelled (biv)anti-MMR Nb upon i.p. injection in 3 weeks tumor-bearing mice. Every dot in the graph corresponds to an individual mouse. Data are presented as Mean ± SEM. D) Whole mount

confocal microscopy analysis of 32 nmoles of AF647-labelled (biv)anti-MMR Nb accumulation in liver, healthy muscle and tumor tissue, upon i.p. injection in 3 weeks tumor-bearing mice. Scale bar: 100 μm.

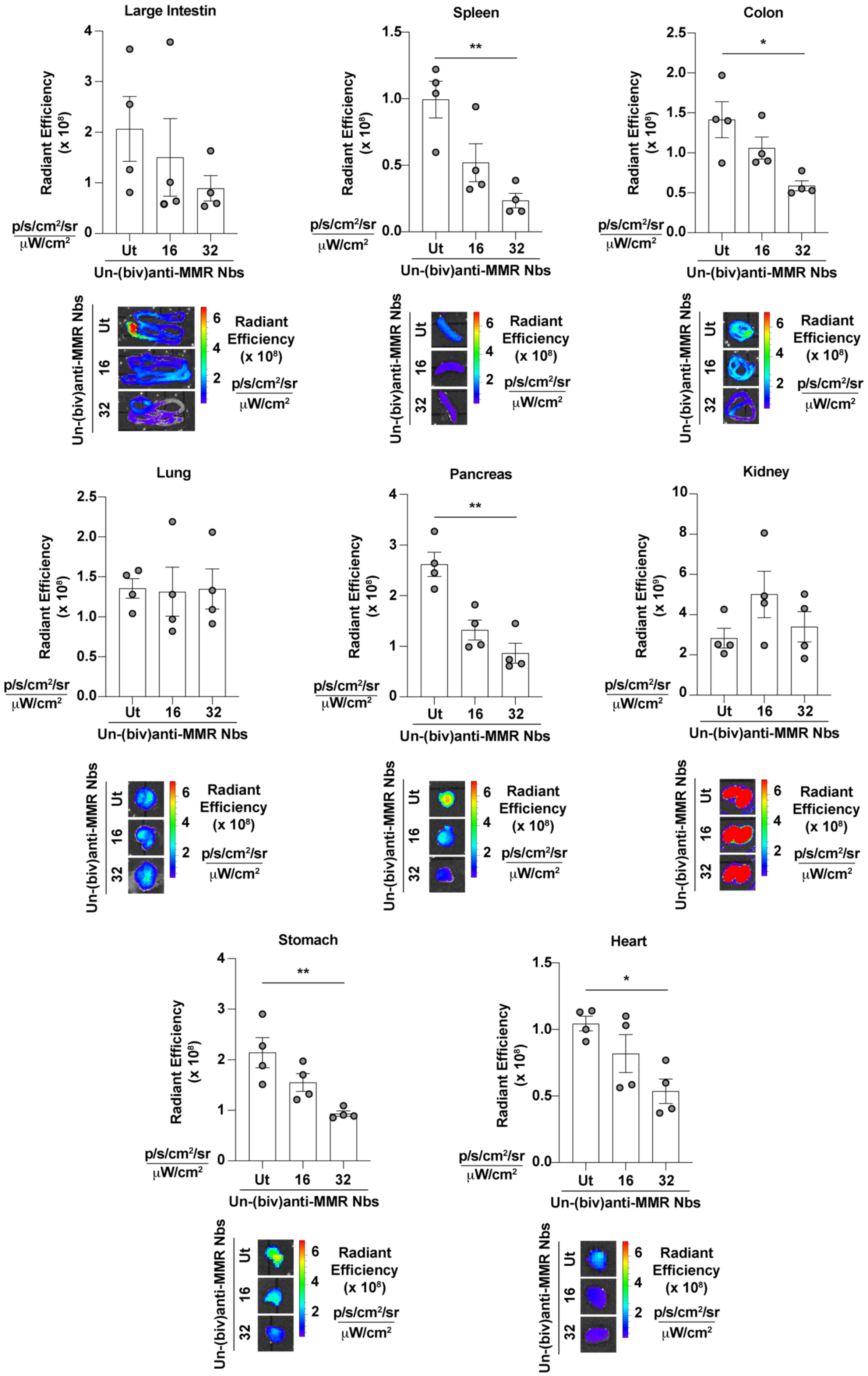

Large Intestin
Spleen
Colon
Lung
Pancreas
Kidney
Stomach
Heart
Radiant Efficiency (x 10⁸)
Radiant Efficiency (x 10⁹)
p/s/cm²/sr / µW/cm²
Ut
16
32
Un-(biv)anti-MMR Nbs
**
*
Radiant Efficiency (x 10⁸)
p/s/cm²/sr / µW/cm²

Supplementary Figure 6. Biodistribution analysis of (m)anti-MMR Nbs upon the administration of a molar excess of unlabelled (biv)anti-MMR Nbs. Representative images of the acquired signal in the different organs are shown. Every dot in the graph corresponds to an individual mouse. Data are presented as Mean ± SEM. Kruskal Wallis plus Dunn's Multiple comparison test, * $p<0.05$, ** $p<0.01$

Movie legends:

Movie1: Intravital microscopy of Cy5-labeled (m)anti-MMR Nbs in liver. Scale Bar: 100 μm.

Movie2: Intravital microscopy of Cy5-labeled (biv)anti-MMR Nbs in liver. Scale Bar: 100 μm.

Movie3: Intravital microscopy of Cy5-labeled (m)anti-MMR Nbs in liver upon the i.p. injection of 32nmol of un-(biv)anti-MMR Nbs. Scale bar: 100 μm.

Movie4: Intravital microscopy of Cy5-labeled (m)anti-MMR Nbs in healthy muscle tissue. Scale Bar: 100 μm.

Movie5: Intravital microscopy of Cy5-labeled (biv)anti-MMR Nbs in healthy muscle tissue. Scale Bar: 100 μm.

Movie6: Intravital microscopy of Cy5-labeled (m)anti-MMR Nbs in healthy muscle tissue upon the i.p. injection of 32nmol of un-(biv)anti-MMR Nbs. Scale bar: 100 μm.

Movie7: Intravital microscopy of Cy5-labeled (m)anti-MMR Nbs in tumor. Scale Bar: 100 μm.

Movie8: Intravital microscopy of Cy5-labeled (biv)anti-MMR Nbs in tumor. Scale Bar: 100 μm.

Movie9: Intravital microscopy of Cy5-labeled (m)anti-MMR Nbs in tumor upon the i.p. injection of 32nmol of un-(biv)anti-MMR Nbs. Scale bar: 100 μm.

Material and Methods:

Surface Plasmon Resonance (SPR)

SPR was performed using a Biacore device (Biacore T200; Cytiva). First, a CM5 sensor chip (Biacore, Series S Sensor Chip CM5; Cytiva) was activated with 1-ethyl-3-(3-dimethylaminopropyl)carbodiimide/N-hydroxysuccinimide (EDC/NHS; Amine Coupling Kit; Cytiva). Subsequently, recombinant murine MMR protein (2535-MM, R&D systems, 5 µg/mL) was immobilized on the chip at pH 5. The SPR measurements were done at 25 °C in HEPES buffered saline (HBS; Cytiva) as a running buffer, with a flow rate of 30 µL/min. For evaluation of the kinetic parameters (association rate k_a, dissociation rate k_d, dissociation constant K_D), the Nbs were injected consecutively in three-fold serial dilutions, from 100 to 0.4 nM. The association step was allowed for 180 s, the dissociation step for 600 s. Regeneration of 20 s at 30 µL/min using 0.1 M glycine at pH 2.5 followed by a stabilization period of 60 s, was performed. The rate kinetic constants were determined by mathematical fitting using the 1:1 binding with drift and RI2 model proposed by the BIACORE Evaluation Software (Cytiva), and the k_d/k_a ratio was used to determine the equilibrium dissociation constant (K_D).

Macro (Fiji_ImageJ) to analyze the amount of Cy5-(m)anti-MMR Nbs within polarized macrophages.

```
run("Open...");
imageTitle=getTitle();
run("Set Measurements...", "area mean limit redirect=None decimal=2");
run("Split Channels");
selectWindow("C3-" + imageTitle);
run("Close");
```

```
selectWindow("C1-" + imageTitle);
rename("Nbs");
selectWindow("C2-" + imageTitle);
run("Duplicate...", " ");
selectWindow("C2-" + imageTitle);
run("Median...", "radius=2");
setAutoThreshold("Default dark");
//run("Threshold...");
setThreshold(10, 255);
waitForUser("set the threshold and press OK, or cancel to exit macro");
setOption("BlackBackground", true);
run("Convert to Mask");
//run("Fill Holes");
run("Create Selection");
run("Set Measurements...", "area mean integrated limit redirect=[Nbs] decimal=2");
run("Measure");
waitForUser("save results, then press ok");
close("Threshold");
close("Results");
run("Close All");
```

Macro (Fiji_ImageJ) to analyse the Nb blood clearance in liver and healthy/tumor tissue

Liver:

```
run("Open...");
imageTitle=getTitle();
run("Split Channels");
selectWindow("C1-" + imageTitle);
rename("Dextran");
run("Duplicate...", "duplicate");
```

```
rename("Dextran_Duplicate");
selectWindow("C2-" + imageTitle);
rename("Nbs");
selectWindow("Dextran");
run("Threshold...");
waitForUser("Set threshold then press ok");
setOption("BlackBackground", true);
run("Convert to Mask", "method=Default background=Dark black");
getDimensions(width, height, channels, slices, frames);
p=frames;
for (n = 1; n <= p; n++) {
	Stack.setFrame(n);

run("Set Measurements...", "mean integrated limit display redirect=Nbs decimal=2");
run("Create Selection");
run("Make Inverse");
roiManager("Add");
run("Measure");
}
waitForUser("Check Analysis, Save Results, then press ok");
close("Threshold");
close("Results");
run("Close All");
close("ROI Manager");
```

Healthy/Tumor:

```
run("Open...");
imageTitle=getTitle();
run("Split Channels");
selectWindow("C2-" + imageTitle);
```

```
rename("Dextran");
run("Duplicate...", "duplicate");
rename("Dextran_Duplicate");
selectWindow("C1-" + imageTitle);
rename("Nbs");
selectWindow("Dextran");
run("Threshold...");
waitForUser("Set threshold then press ok");
setOption("BlackBackground", true);
run("Convert to Mask", "method=Default background=Dark black");
getDimensions(width, height, channels, slices, frames);
p=frames;
for (n = 1; n <= p; n++) {
	Stack.setFrame(n);


run("Set Measurements...", "mean integrated limit display redirect=Nbs decimal=2");
run("Create Selection");
//run("Make Inverse");
roiManager("Add");
run("Measure");
}
waitForUser("Check Analysis, Save Results, then press ok");
close("ROI Manager");
close("Results");
for (n = 1; n <= p; n++) {
	Stack.setFrame(n);
run("Set Measurements...", "mean integrated limit display redirect=Dextran_Duplicate decimal=2");
run("Create Selection");
//run("Make Inverse");
roiManager("Add");
```

```
run("Measure");
}
waitForUser("Check Analysis, Save Results, then press ok");
close("Threshold");
close("Results");
run("Close All");
close("ROI Manager");
```
